# The Molecular Mechanism of Ethylene-Mediated Root Hair Development Induced by Phosphate Starvation

**DOI:** 10.1371/journal.pgen.1006194

**Published:** 2016-07-18

**Authors:** Li Song, Haopeng Yu, Jinsong Dong, Ximing Che, Yuling Jiao, Dong Liu

**Affiliations:** 1 Ministry of Education Key Laboratory of Bioinformatics, Center for Plant Biology, School of Life Sciences, Tsinghua University, Beijing, China; 2 State Key Laboratory of Plant Genomics, National Centre for Plant Gene Research, Institute of Genetics and Developmental Biology, Chinese Academy of Sciences, Beijing, China; University of California Berkeley, UNITED STATES

## Abstract

Enhanced root hair production, which increases the root surface area for nutrient uptake, is a typical adaptive response of plants to phosphate (Pi) starvation. Although previous studies have shown that ethylene plays an important role in root hair development induced by Pi starvation, the underlying molecular mechanism is not understood. In this work, we characterized an *Arabidopsis* mutant, *hps5*, that displays constitutive ethylene responses and increased sensitivity to Pi starvation due to a mutation in the ethylene receptor ERS1. *hps5* accumulates high levels of EIN3 protein, a key transcription factor involved in the ethylene signaling pathway, under both Pi sufficiency and deficiency. Pi starvation also increases the accumulation of EIN3 protein. Combined molecular, genetic, and genomic analyses identified a group of genes that affect root hair development by regulating cell wall modifications. The expression of these genes is induced by Pi starvation and is enhanced in the *EIN3*-overexpressing line. In contrast, the induction of these genes by Pi starvation is suppressed in *ein3* and *ein3eil1* mutants. EIN3 protein can directly bind to the promoter of these genes, some of which are also the immediate targets of RSL4, a key transcription factor that regulates root hair development. Based on these results, we propose that under normal growth conditions, the level of ethylene is low in root cells; a group of key transcription factors, including RSL4 and its homologs, trigger the transcription of their target genes to promote root hair development; Pi starvation increases the levels of the protein EIN3, which directly binds to the promoters of the genes targeted by RSL4 and its homologs and further increase their transcription, resulting in the enhanced production of root hairs. This model not only explains how ethylene mediates root hair responses to Pi starvation, but may provide a general mechanism for how ethylene regulates root hair development under both stress and non-stress conditions.

## Author Summary

Phytohormone ethylene has previously been known to play an important role in mediating root hair development induced by phosphate starvation; however, the underlying molecular mechanism is not understood. Using combined molecular, genetic, and genomic approaches, we identify a group of genes that affect root hair development by regulating cell wall modifications. Pi starvation increase the stability of EIN3 protein, a key component in the ethylene signaling pathway. The expression of the identified root hair-related genes is enhanced in the *EIN3*-overexpressing line, but suppressed in the *ein3* mutant. Furthermore, EIN3 protein directly binds to the promoter of these genes which are also targeted by a key transcription factor that regulates root hair development. This work not only explains how ethylene mediates root hair responses to phosphate starvation, but may provide a general mechanism for how ethylene regulates root hair development under both stress and non-stress conditions.

## Introduction

As an essential macronutrient, phosphorus (P) plays vital roles in plant growth, development, and metabolism. P not only serves as structural elements of nucleic acids and phospholipids, but is also involved in many important biological processes, including photosynthesis, oxidative phosphorylation, regulation of enzymatic activities, and cell signaling. Using the Pi transporters localized on the root surface, plants take up P from the soil in the form of inorganic phosphate (Pi) [1]. When plants are exposed to Pi deficiency, they activate an array of adaptive responses to cope with this nutritional stress. These responses involve developmental, biochemical, and physiological changes, including the reprogramming of root development; increased activities of high affinity Pi transporters; the induction and secretion of acid phosphatases, RNases, and organic acids; and the accumulation of anthocyanin and starch [2,3].

The Pi starvation-induced changes in root development include the inhibition of primary root growth and the increased production of lateral roots and root hairs [4]. Root hairs, which are tubular outgrowths of root epidermal cells, account for a large portion of the root surface area involved in water and nutrient uptake [5]. For rye plants grown under Pi starvation, root hairs are responsible for nearly 60% of the Pi absorbed [6]. In Pi deficient-*Arabidopsis* plants, root hairs represent 91% of the total root surface area [7]. Under low Pi conditions, wild-type (WT) *Arabidopsis* plants acquire more Pi than mutants that are defective in root hair formation [8]. Root hairs also modify the rhizosphere by exuding large amounts of organic acids, enzymes, mucilage, and secondary metabolites [9]. The enhanced growth of root hairs has been thought to be the earliest root morphological response to Pi starvation [8]. Low Pi availability increases root hair length by increasing root hair growth rate and growth duration [10]. The increase in root hair density in Pi deficient-plants is due to the increase in trichoblast file number, the reduction in trichoblast length, and/or the increase in the percentage of trichoblast cells that form root hairs [11,12].

Pi starvation alters ethylene biosynthesis and sensitivity in plants (for a review, see [13]). Ethylene is a gaseous phytohormone that has many functions in plant growth and development and in plant responses to stress conditions. In *Arabidopsis*, ethylene is perceived by five receptors located in endoplasmic reticulum (ER) membranes [14]. In the absence of ethylene, ethylene receptors activate CTR1 (CONSTITUTIVE TRIPL1 RESPONSE1, a Raf1-like kinase), which subsequently suppresses the function of its downstream target EIN2 (ETHYLENE INSENSTIVE2). When ethylene binds to its receptors, it inactivates CTR1, which causes the translocation of a C-terminal fragment of EIN2 (EIN2-C’) into the nucleus. In the nucleus, EIN2-C’ increases the protein levels of EIN3 (ETHYLENE INSENSITIVE3) and its closest homolog EIL1 (ETHYLENE INSENSITIVE3-LIKE1), two key transcription factors of the ethylene signaling pathway. EIN3 and EIL1 then turn on the transcription of a battery of downstream target genes, which initiate a diverse array of plant responses to internal and external cues.

Ethylene has long been known to be a positive regulator of root hair development [15,16]. It acts downstream of the key transcription factors GL2 and RHD6 to promote root hair formation [17]. Previous reports have also indicated that ethylene plays an important role in Pi starvation-induced root hair development [7,12]. Application of inhibitors of ethylene biosynthesis or action reduces Pi starvation-induced root hair development in *Arabidopsis*. Similarly, the root hair responses to Pi starvation are significantly attenuated in ethylene-insensitive mutants of *Arabidopsis* [12]. To date, however, the molecular mechanism of how ethylene mediates Pi starvation-induced root hair development remains unknown.

In this report, we characterize an *Arabidopsis* mutant, *hps5*, that carries a mutation in the ethylene receptor ERS1. *hps5* accumulates higher levels of EIN3 protein than the WT under both Pi sufficiency and deficiency. Pi starvation also increases the accumulation of EIN3. By combining genetic, molecular, and transcriptomic approaches, we first identify a group of ethylene-regulated genes involved in Pi starvation-induced root hair development. We show that EIN3 directly binds to the promoters of genes that are the targets of a key transcription factor that regulates root hair development. Finally, we propose a working model of how ethylene mediates root hair responses to Pi starvation. This model may also provide a general mechanism for how ethylene regulates root hair development under both stress and non-stress conditions.

## Results

### *hps5* has enhanced root hairs production

**Fig 1:**
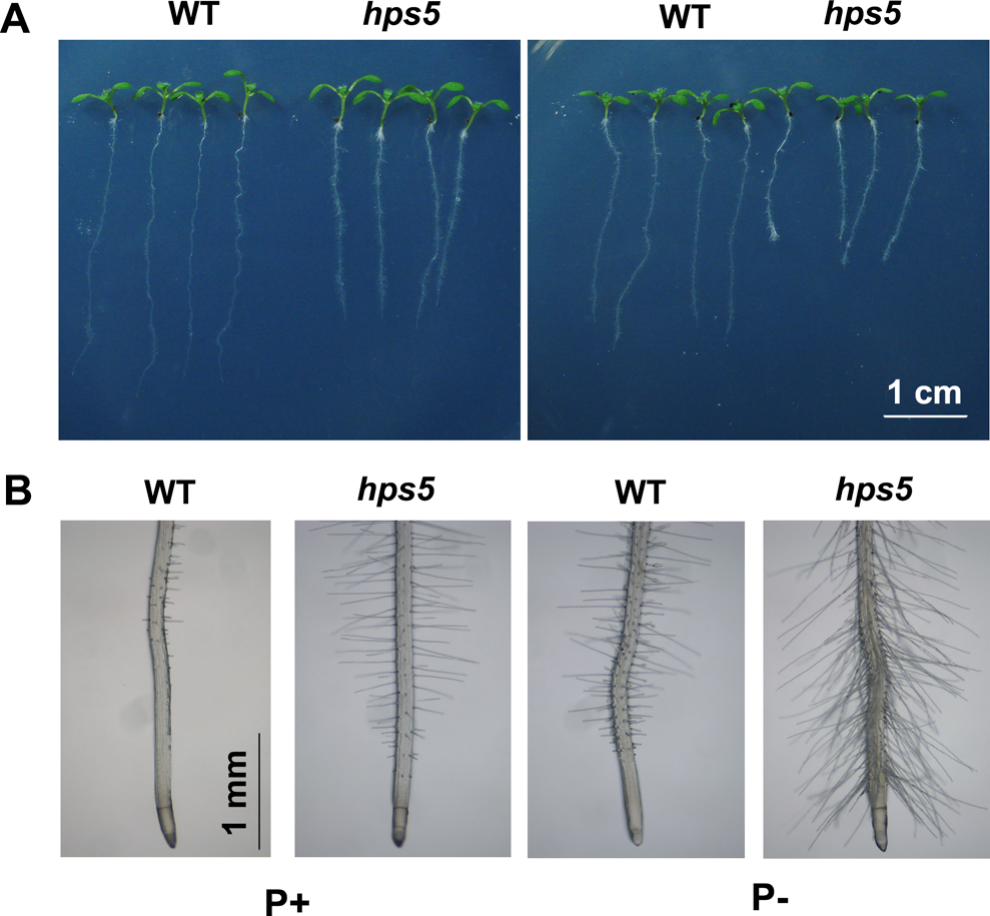
Growth characteristics of WT and *hps5* mutant seedlings. (A), Morphology of 7-day-old seedlings of the WT and *hps5* grown on P+ and P- media. (B), A close view of the root tips of the WT and *hps5*.

To identify the molecular components involved in Pi starvation-induced root hair production, we performed a large-scale screen for the *Arabidopsis* mutants with enhanced root hair production under Pi deficiency. One such mutant, *hps5* (*h**ypersensitive to* *P**i* *s**tarvation5*), was obtained through this screen. Both root hair length and root hair density were increased in *hps5* compared to the WT under Pi deficiency (P-) (Fig 1A and 1B). Root hair production was also greater in *hps5* than in the WT under Pi sufficiency (P+).

In addition to producing more root hairs, *hps5* also exhibited altered primary and lateral root growth. Pi starvation inhibited primary root growth and promoted lateral root formation in the WT (Fig 1). At 7 days after germination (DAG), the primary root was about 30% shorter for *hps5* than for the WT on P+ medium but was about 50% shorter on P- medium (S1A Fig). At 9 DAG, lateral root density was less for *hps5* than for the WT on P+ medium but was similar on P- medium (S1B Fig).

### *hps5* has altered sensitivity in other Pi starvation responses

Next, we examined other Pi responses of *hps5*. Induction and secretion of acid phosphatases (APases) is a hallmark response of plants to Pi deficiency [18]. The Pi starvation-induced APases function in Pi remobilization and recycling in old tissues, and in the utilization of organophosphate compounds in the rhizosphere. The APase activity on the root surface can be detected by histochemical staining using the APase substrate BCIP (5-bromo-4-chloro-3-indolyl-phosphate) [19]. Cleavage of BCIP by APases produces a blue precipitate. Using this method, we found that *hps5* had a stronger blue staining on its root surface than the WT under Pi deficiency (S2A Fig). Under Pi sufficiency (P+), *hps5* roots also exhibited a light blue staining while WT roots were white (S2A Fig). The enhanced root surface-associated APase activity in *hps5* was confirmed by quantitative analysis (S2B Fig).

The expression of eight Pi starvation-induced marker genes was analyzed. These genes encode two non-coding transcripts, At4 and IPS1; an RNase, RNS1; a microRNA, miRNA399D; two APases, ACP5 (AtPAP17) and AtPAP10; and two high-affinity Pi transporters, AtPT1 (Pht1;1) and AtPT2 (Pht1;4) [20]. At 7 DAG, the expression of all of these marker genes, except *AtPT1* and *AtPT2*, was significantly higher in *hps5* seedlings than in the WT under Pi deficiency (S3 Fig).

Accumulation of anthocyanin in leaves is another characteristic response of plants to Pi starvation. Induction of anthocyanin is thought to protect the chloroplast membranes under Pi deficiency [3]. At 10 DAG on P- medium and P- medium with 10–100 μM Pi, the leaves of the seedlings had turned purple but the intensity of the purple was greater in the WT than in *hps5* (S4 Fig). These results indicated that *hps5* was less sensitive to Pi starvation-induced anthocyanin accumulation in leaves.

The total P and cellular Pi contents in shoots and roots of 9-day-old seedlings did not significantly differ between *hps5* and the WT under either P+ or P- conditions (S5 Fig).

### *hps5* contains a point mutation in the ethylene receptor ERS1

**Fig 2:**
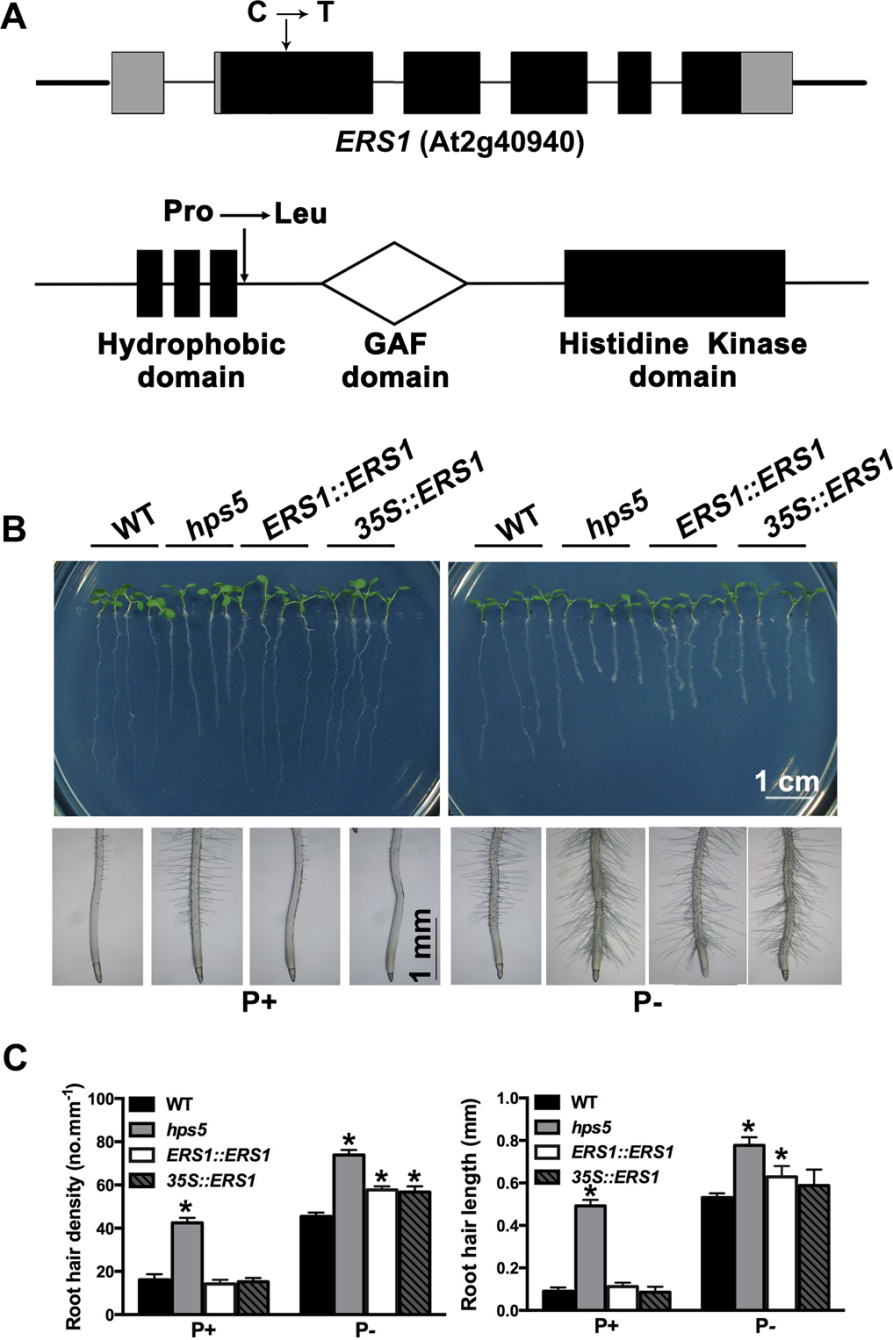
Molecular identification and genetic complementation of the *HPS5* gene. (A), Diagrams showing the positions of the mutations in the *ERS1* gene and ERS1 protein. Top: the structure of the *ERS1* gene. Black box: exons; grey box: 5’ and 3’ UTRs; thin line: introns; thick line: flanking sequences surrounding the *ERS1* gene. Bottom: the structure of the ERS1 protein showing the signature domains. (B), Morphology of 7-day-old seedlings and root tips of the WT, *hps5*, and complementation lines grown on P+ and P- media. (C), Quantitative analyses of root hair density and root hair length of various lines. Values represent means ± SD of three replicates. Asterisks indicate a significant difference from the WT (*t*-test, P < 0.05). For each measurement, 15–20 seedlings were used.

When *hps5* was backcrossed to the WT, all of the F_1_ plants showed WT phenotypes. The F_2_ progeny derived from the selfed F_1_ plants segregated into WT and mutant phenotypes in a ratio of about 3:1 (203:62), indicating that the mutant phenotypes were caused by a single recessive mutation. Using a map-based cloning approach (S6 Fig), we found a C to T mutation in the second exon of the *ERS1* gene (At2g40940) in *hps5* (Fig 2A). This mutation converted a proline to a leucine at the position of 110 of the ERS1 protein. Depending on the program used for prediction, the mutated proline residue was predicted to be located at the third or fourth amino acid position after the transmembrane domain III [21, 22]. This amino acid residue is highly conserved in the ERS1 orthologs in all major crops (S7 Fig), suggesting that it is important for the receptor function of ERS1.

To confirm that the mutated *ERS1* gene was responsible for the *hps5* phenotypes, we introduced the genomic sequence of WT *ERS1* back into *hps5* plants under the control of the cauliflower mosaic virus (CaMV) 35S promoter or *ERS1*’s own promoter. Both gene constructs (*CaMV 35S*::*ERS1* and *ERS1*::*ERS1*) could fully complement the *hps5* phenotypes on P+ medium, in terms of reduced primary root growth, increased root hair production, and enhanced root-associated APase activity (Fig 2B and S8 Fig). On P- medium, however, the two gene constructs could only partially rescue these mutant phenotypes (Fig 2B and 2C, S8 Fig). Partial complementation under P- condition may result from interference between the mutated ERS1 protein and the other ethylene receptors that is not fully reversed by the WT ERS1 transgenic protein.

### *hps5* seedlings display constitutive ethylene responses

**Fig 3:**
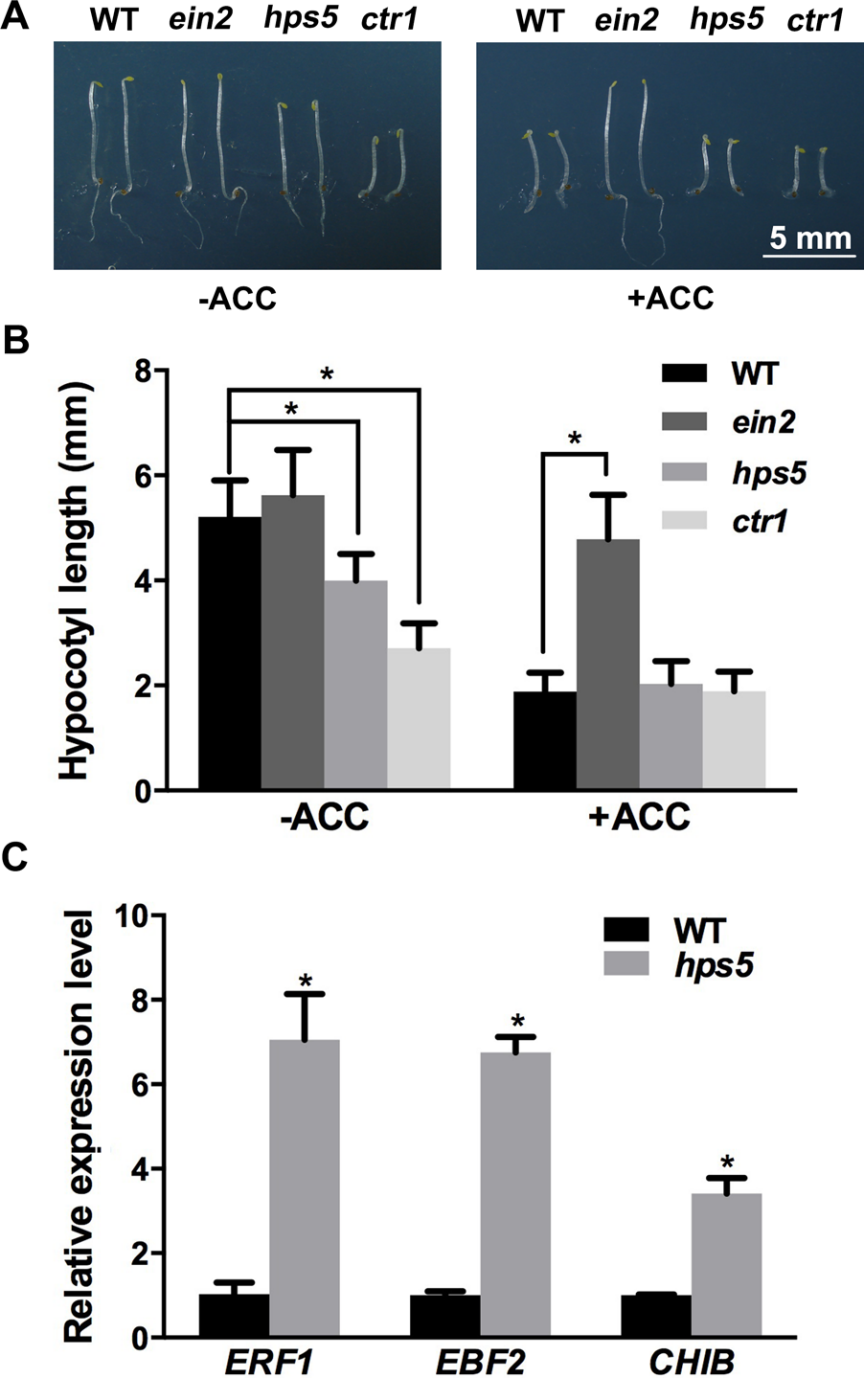
Ethylene responses of the WT and various mutants. (A), Morphology of 4-day-old etiolated seedlings of the WT, *ein2*, *hps5*, and *ctr1* grown in the dark in the absence or presence of 1 μM ACC. (B), The length of the hypocotyls of the seedlings in (A). Values represent means ± SD of three replicates. (C), Relative expression as determined by qPCR of the ethylene-responsive marker genes in the 7-day-old WT and *hps5* seedlings. Values are means ± SD of three technical replications in one experiment that was performed three times with similar results. In (B) and (C), Asterisks indicate a significant difference from the WT (*t*-test, P < 0.05).

When treated with ethylene in the dark, *Arabidopsis* seedlings display a characteristic “triple response”, i.e., an inhibition of root and hypocotyl growth, a radial swelling of the root and hypocotyl, and an increase in the curvature of the apical hook [23]. When grown in soil, WT *Arabidopsis* plants treated with ethylene or the mutant with constitutive ethylene response are small in stature. We compared these growth characteristics among the WT and the mutants *hps5*, *ein2*, and *ctr1*. *ein2* is completely insensitive to ethylene [24], and *ctr1* displays constitutive ethylene responses [25]. When grown in the dark in the absence of ACC (1-aminocyclopropane-1-carboxylic acid), a precursor of ethylene biosynthesis, the WT and *ein2* had a long hypocotyl and a mild apical hook whereas *ctr1* had a much shorter hypocotyl and an exaggerated apical hook (Fig 3A and 3B). For *hps5*, the length of the hypocotyl and the curvature of the apical hook were intermediate relative to the WT and *ctr1*. When treated with 1 μM ACC, the WT, *hps5*, and *ctr1* produced shorter hypocotyls and apical hooks with increased curvature, while *ein2* did not respond to ACC. Under normal growth conditions, *hps5* also showed higher expression of the ethylene-responsive genes *ERF1*, *EBF2*, and *CHIB* [26] than the WT (Fig 3C). Together, these results indicate that *hps5* seedlings display a moderate constitutive ethylene response.

The size and morphology of adult plants grown in soil did not significantly differ between *hps5* and the WT (S9 Fig).

### The *hps5* mutation has a “dominant-negative” effect

**Fig 4:**
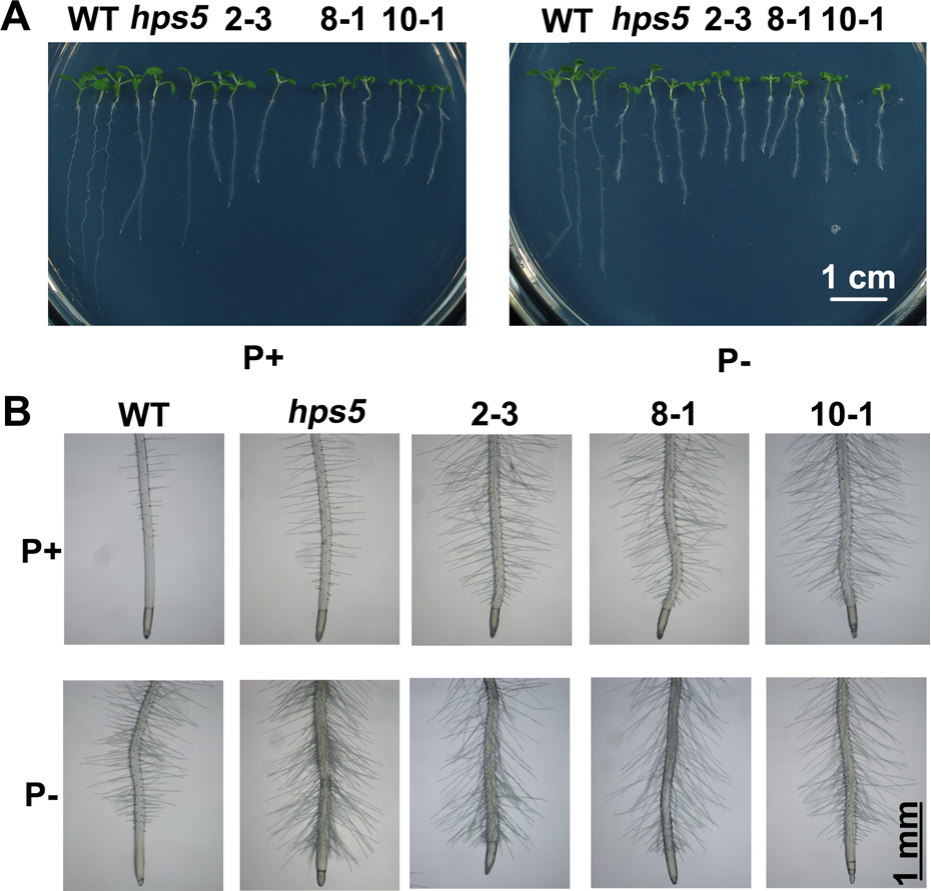
The dominant-negative effect of the *hps5* mutation. (A), Morphology of 7-day-old seedlings of the WT, *hps5*, and three *35S*::*mERS1* transgenic lines grown on P+ and P- media. (B), A close view of the root tips of the seedlings in (A). In (A) and (B), the numbers at the top are the names of the transgenic lines.

**Fig 5:**
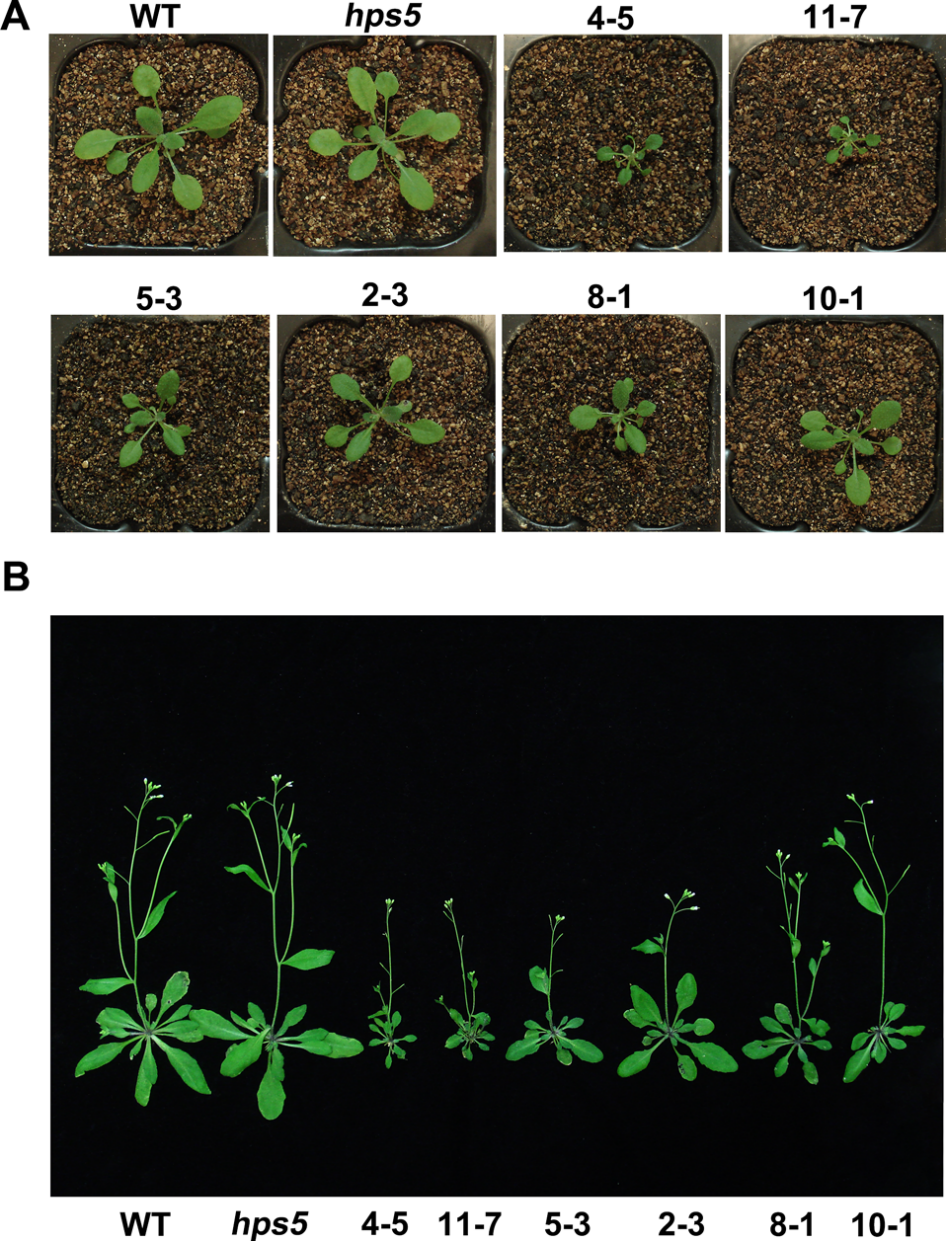
Morphology of the WT, *hps5*, and six *35S*::*mERS1* transgenic lines grown in soil. (A), 25-day-old plants; (B), 37-day-old plants. In (A) and (B), the numbers at the top are the names of the transgenic lines.

*Arabidopsis* has five ethylene receptors: ETR1, ERS1, ETR2, EIN4, and ERS2 [14]. Because of functional redundancy, the loss-of-function mutation in a single receptor gene does not cause any mutant phenotypes. In contrast, a point mutation that abolishes the binding of ethylene to any of these five receptors makes plants insensitive to ethylene [27,28]. Because the point mutation in *ERS1* caused constitutive ethylene responses, we suspected that this mutation had a “dominant-negative” effect, an effect that interferes with the functions of other ethylene receptors. To test this hypothesis, we isolated the mutated *ERS1* gene (*mERS1*) from *hps5* and introduced it into WT plants under the control of the *CaMV 35S* promoter. When grown in soil, most of 25 primary transformants were smaller than the WT. Overexpression of the *mERS1* gene was confirmed by qPCR in six selected transgenic lines (S10 Fig). We then generated homozygote plants for the transgene (*35S*::*mERS1*) for these lines. When grown on P+ and P- media, the seedlings of these lines showed strong inhibition of primary root growth (Fig 4A and S11 Fig), increased root hair production (Fig 4B), and enhanced root-associated APase activity (S12A and S12B Fig), mimicking the phenotypes of *hps5*. When grown in soil, the size of these six transgenic lines varied, but they were all smaller than the WT and *hps5* (Fig 5). These results suggested that the mutated ERS1 protein might interfere with the functions of WT ERS1 and other ethylene receptors in the transgenic plants.

### *hps5* mutation and Pi starvation increase the accumulation of EIN3 protein

**Fig 6:**
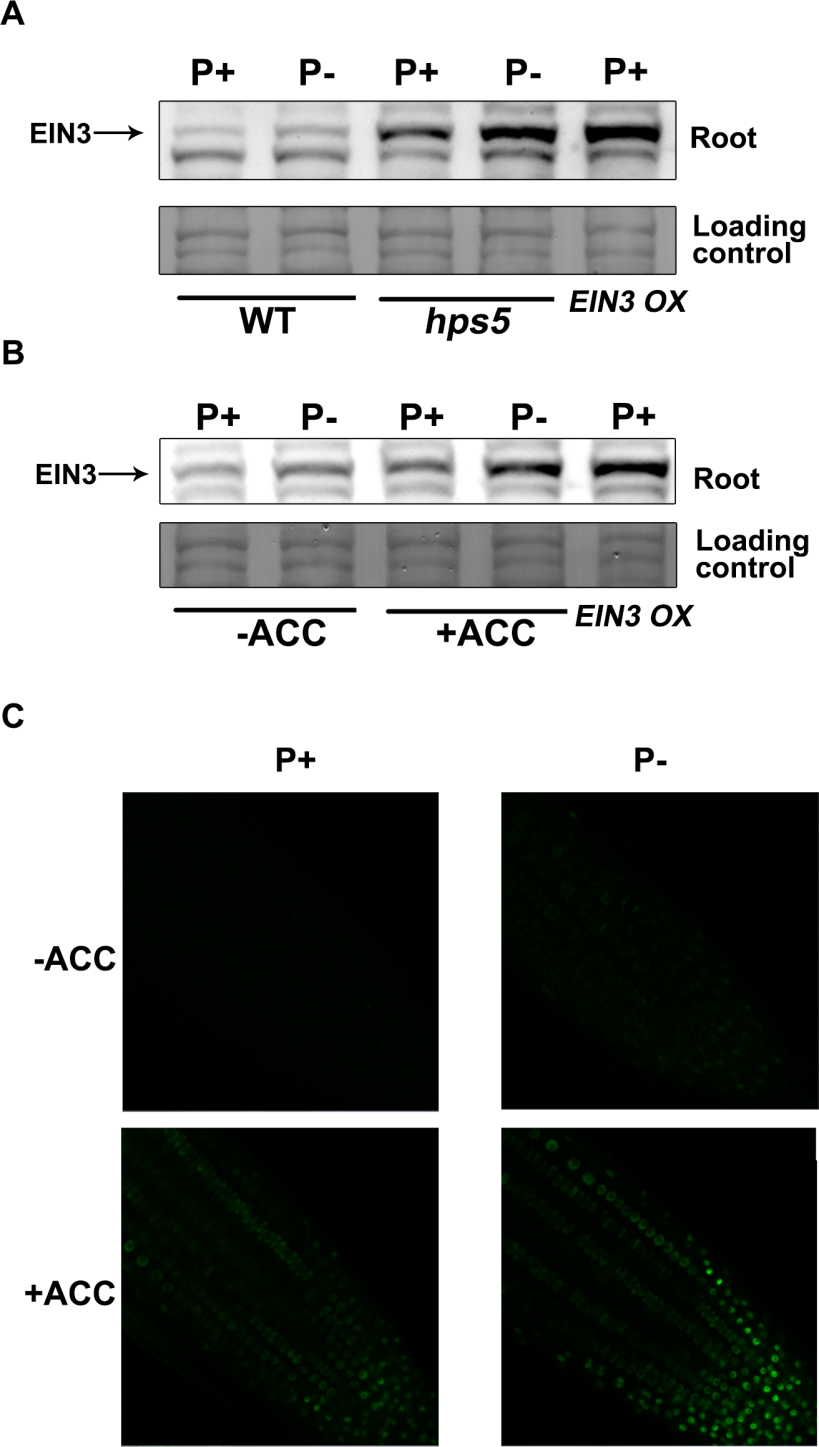
Accumulation of EIN3 protein in WT, *hps5*, and ACC-treated seedlings grown under P+ and P- conditions. (A) Western blot analysis of EIN3 proteins in WT and *hps5* seedlings. (B) Western blot analysis of EIN3 proteins in WT plants treated or not treated with 10 μM ACC. In (A) and (B), the antibodies raised against native EIN3 protein were used. The proteins extracted from the seedlings of the *EIN3 OX* line were used as the positive control of EIN3 protein. (C) Confocal analysis of the accumulation of EIN3-GFP in the root tips of 7-day-old *35S*::*EIN3-GFP* seedlings grown under various conditions.

To investigate how ethylene signaling is enhanced in *hps5*, we compared the mRNA and protein levels of EIN3 in roots of the WT and *hps5*. qPCR analysis indicated that the mRNA level of *EIN3* in the WT and *hps5* was similar under both P+ and P- conditions (S13A Fig); as revealed by Western blot, however, the level of EIN3 protein was higher in *hps5* than in the WT (Fig 6A). We then examined the effects of Pi starvation on the transcription and protein accumulation of EIN3. Like *hps5* mutation, Pi starvation did not alter the level of *EIN3* mRNA (S13B Fig) but increased the accumulation of EIN3 protein (Fig 6B).

Ethylene or ACC treatment is known to induce the accumulation of EIN3 protein by blocking its degradation as mediated by the F-box proteins EBF1 and EBF2 [26]. By Western blot analysis, we found that the ACC-induced accumulation of EIN3 protein in roots was further enhanced by Pi starvation (Fig 6B). This result was corroborated by the confocal analysis of the expression of EIN3-GFP fusion proteins in the transgenic line expressing the *EIN3-GFP* fusion gene under the control of the 35S promoter (Fig 6C).

### EIN3 and EIL1 are involved in Pi starvation-induced root hair development

**Fig 7:**
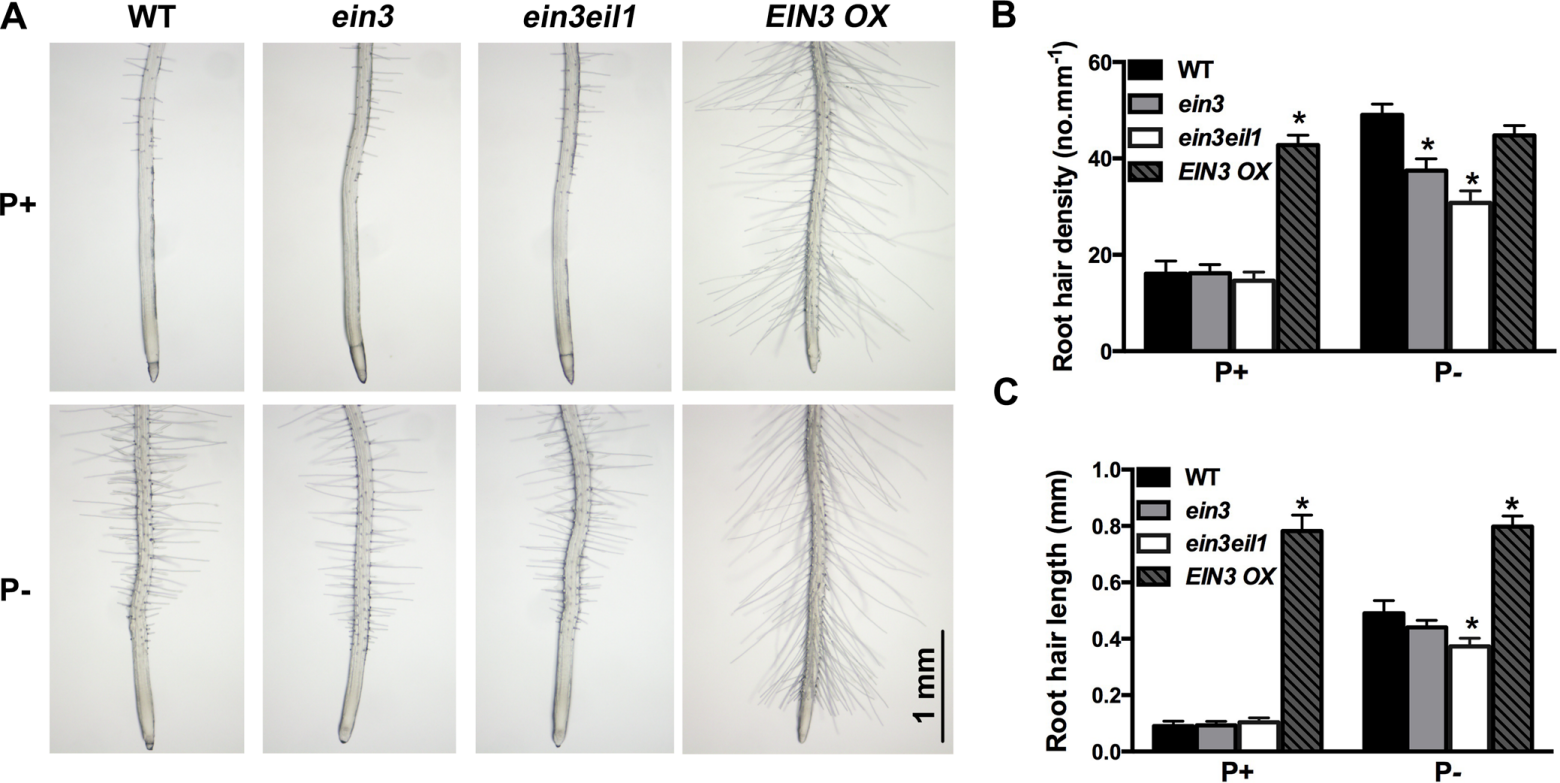
Root hair phenotypes of the WT, *ein3*, *ein3eil1*, and *EIN3 OX* lines. (A), Root hair patterns of the first 3 mm of the root tips of 7-day-old WT, *ein3*, *ein3eil1*, and *EIN3 OX* seedlings grown on P+ and P- media. (B) and (C), Quantitative analyses of root hair density and length of the WT, *ein3*, *ein3eil1*, and *EIN3 OX* seedlings in (A). Values represent means ± SD of three replicates. Asterisks indicate a significant difference from the WT (*t*-test, P < 0.05).

Because Pi starvation increases the accumulation of EIN3 protein, we next asked whether EIN3 and its closest homolog, EIL1, are directly involved in the Pi starvation-induced root hair development. To answer this question, we analyzed the root hair phenotypes of *ein3* and *ein3eil1* mutants and of an *EIN3*-overexpressing line (*EIN3 OX*). On P+ medium, the root hair density and root hair length of *ein3* and *ein3eil1* did not significantly differ from those of the WT (Fig 7). On P- medium, root hair density was lower for both *ein3* and *ein3eil1* than for the WT but root hair length was reduced only for *ein3eil1*. In contrast, both root hair length and root hair density were greatly increased in *EIN3 OX* relative to the WT. These results indicate that EIN3 and EIL1 are involved in Pi starvation-induced root hair production.

### Identification of the target genes of the ethylene signaling involved in Pi starvation-induced root hair development

To identify the downstream targets of the ethylene signaling involved in Pi starvation-induced root hair development, we compared the transcriptomes of the WT, *hps5*, *ein3*, and *ein3eil1*. The cDNA libraries were constructed using the total RNAs isolated from 7-day-old seedlings grown on P+ or P- medium. For each combination of genotype and Pi treatment, two biological replicates were used. Log_2_≥1 or ≤-1 (2-fold change in expression levels) and FDR (false discovery rate) ≤ 0.01 were used as the cut off for selection of the genes whose expression levels significantly differed from that of P+ WT. The transcriptomic data indicated that the expression of Pi-responsive marker genes, such as *Pht1;1*, *Pht1;4*, *ACP5*, *At4*, and *SPX1*, was upregulated in P- WT compared to P+ WT (S1 Table), indicating that the experimental conditions for RNA-seq analyses were proper.

We first compared the expression profiles of *hps5* and the WT grown on P+ medium. Surprisingly, based on our selection criteria, there were only 61 genes whose expression was upregulated and 43 genes whose expression was downregulated in *hps5* relative to the WT (S2 Table). This indicated that the effect of *hps5* mutation on gene expression at the genomic level was relatively mild, which was consistent with the absence of developmental abnormality for adult *hps5* plants grown in soil. Among the 61 upregulated genes, there were seven RHS (Root Hair-Specific) genes [29], including *RHS2* (a MATE [multidrug and toxin efflux] family protein), *RHS12* (a pectinesterase), *RHS13* (an extensin-like protein), *RHS14* (a pectate lyase), *RHS15* (a glycerol-3-phosphate permease, G3PP2), *RHS18* (a peroxidase), and *RHS19* (a peroxidase). The induction level of *RHS16* (a leucine-rich repeat protein kinase) was also close to 2-fold (Log_2_ = 0.97). *RHS2*, *RHS16*, and *RHS18* have previously been shown to be involved in root hair elongation or branching [29]. In addition, the upregulated genes included two xyloglucan endotransglucosylases (XTH12 and XTH14), two other peroxidases (PER13a and At3g49960), and one pectin methyltransferase inhibitor. XTH12 and XTH14 are the two most closely related members of the XTH family in *Arabidopsis* and are mainly expressed in roots [30]. The enzymatic activity of the recombinant XTH14 protein has been demonstrated in *vitro* and this protein can affect cell elongation and root hair morphology [31]. In addition, there were 16 cell wall proteins, including 10 proline-rich extensins (EXT2, EXT7, EXT10, EXT11, EXT12, EXT13, EXT14, EXT15, EXT16, and EXT18), two proline-rich proteins (PRP1 and PRP3), two leucine-rich repeat extensins (LRX1 and LRX2), one FASCICLIN-like arabinogalactan protein (FLA6), and one arabinogalactan protein (AGP3). Among them, the knockout mutants for *EXT7*, *EXT11*, *EXT14*, *EXT16*, and *EXT18* [32], and *LRX1* and *LRX2* [33] have abnormal root hair initiation, elongation, and morphologies. The expression of *PRP3* and *AGP3* is regulated by root hair-specific developmental pathways [34,35]. Besides, *COW1* (*CAN OF WORMS1*), *AHA7* (*Arabidopsis* H+-ATPase 7), and *ARGOS*-like (*ARL*) genes were upregulated in *hps5*. *COW1* encodes a Sec14p-like phosphatidylinositol transfer protein [36]. The loss-of-function mutants of *COW1* and its homolog in rice, *OsSNDP1*, produced short root hairs with irregular shapes [36,37]. The *aha7* T-DNA insertional mutant has reduced root hair density under both normal and Fe-deficient conditions [38]. Because the initiation and elongation of root hairs are associated with a localized decrease in apoplastic pH [39], AHA7 may regulate root hair density by modifying local pH levels. ARL functions in cell expansion-dependent organ growth [40]. Reduced expression or overexpression of *ARL* results in decreased or increased cell size in *Arabidopsis* cotyledons, leaves, and other lateral organs.

Notably, five of the genes that were upregulated in *hps5* are involved in jasmonate (JA) biosynthesis or signalling. These genes encode phospholipase A 2A (PLA2A) [41], sulfotransferase 2A (ST2A) [42], lipoxygenase 2 (LOX2) [43], salt tolerance zinc finger (ZAT10) [44], and octadecanoid-responsive AP2/ERF59 (ORA59) [45]. This suggests that enhanced ethylene signaling might upregulate JA biosynthesis and signalling.

None of the 43 genes that were downregulated in P+ *hps5* relative to P+ WT was significantly enriched in any functional category, except that five of the genes encoding bifunctional inhibitor/lipid-transfer protein/seed storage 2S albumin superfamily proteins (S2 Table). The biological relevance of the downregulation of these genes is unknown.

**Fig 8:**
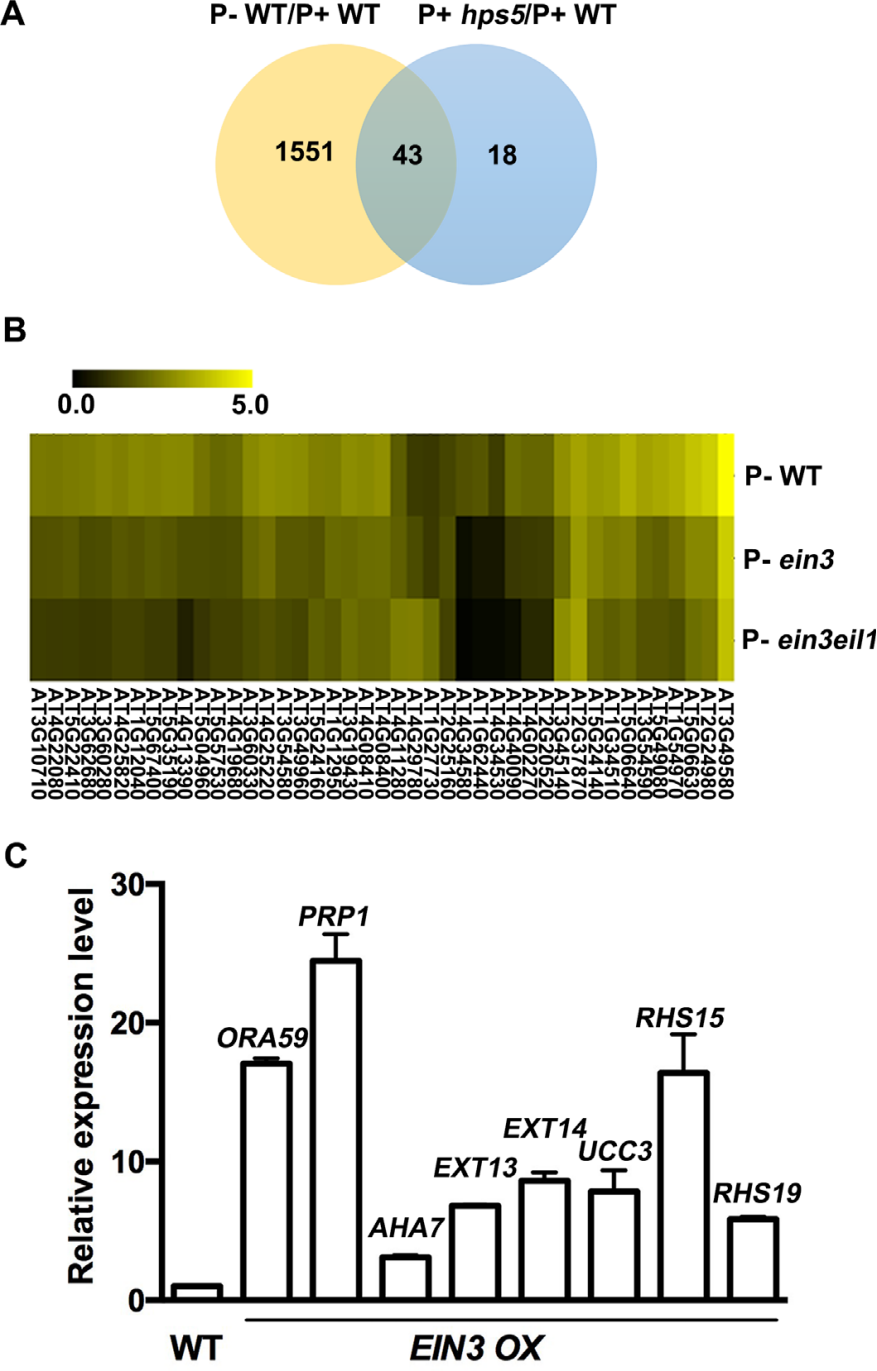
Identification of ethylene and Pi starvation-regulated genes by RNA-seq analyses. (A), A Venn diagram showing the number of transcripts upregulated in P+ *hps5* (blue) and P- WT (yellow), respectively, and the number of transcripts which are upregulated in both P+ *hps5* and P- WT (grey). (B), Hierarchical clusters displaying the Pi starvation-induced genes in the WT, *ein3*, and *ein3eil1* plants. (C), Expression of eight genes in *EIN3 OX* relative to the WT as determined by qPCR. The expression of each of these genes in the WT was set to 1.0.

We then analysed the transcriptomes of the Pi deficient-WT (P- WT) seedlings. Under our experimental conditions, 1,594 genes were upregulated (Fig 8A) and 673 genes were downregulated in the WT in response to Pi deficiency (S1 Table). Among the 61 upregulated genes in P+ *hps5* (S2 Table), 43 were also upregulated by Pi starvation in the WT, and these included the seven *RHS*s and the genes encoding most cell wall proteins and cell wall modifying enzymes (Fig 8A, Table 1). The accuracy of the RNA-seq experiments was verified by qPCR analysis of six selected genes (S14 Fig). We then examined the expression of these 43 genes in P- *ein3* and P- *ein3eil1* (S3 and S4 Tables). The expression levels of 37 genes were lower in P- *ein3* than in P- WT (Fig 8B, Table 1). And, the expression of most of these 37 genes was further downregulated in P- *ein3eil1* (Fig 8B, Table 1). These RNA-seq results were also validated by qPCR analysis of six selected genes (S15 Fig). Furthermore, we randomly selected eight genes for qPCR analysis and found that their expression in the *EIN3 OX* line was significantly elevated under normal growth conditions (Fig 8C).

**Table 1:** The genes whose expression is increased in both P+ *hps5* and P- WT.

AGI Code	Gene name	Gene description	Fold Change (Log2)			
			P+ *hps5*	P- WT	P- *ein3*	P- *ein3eil1*
AT2G24980	*EXT7*	Extensin	2.51	4.05	2.69	2.33
AT5G06630	*EXT13*	Extensin	2.32	3.85	2.68	2.18
AT1G54970*	*PRP1*	Proline-rich protein	2.30	3.47	2.22	1.56
AT3G49580	*LSU1*	Transcript induced by low sulfur	2.25	5.79	4.01	3.82
AT5G49080	*EXT16*	Extensin	2.25	3.30	1.90	1.65
AT5G06640*	*EXT14*	Extensin	2.12	3.47	2.54	2.03
AT4G25220*	*RHS15*	Glycerol-3-phosphate permease	2.12	2.89	2.21	1.57
AT4G08400	*EXT11*	Extensin	1.89	2.86	2.16	2.04
AT4G19680*	*IRT2*	Iron regulated transporter	1.86	2.15	1.53	1.26
AT1G34510*	*PER13a*	Peroxidase	1.84	3.12	2.45	1.82
AT2G20520*	*FLA6*	FASCICLIN-like arabinogalactan	1.74	2.04	1.24	-
AT4G08410*	*ETX12*	Extensin	1.74	2.71	2.15	2.01
AT5G35190	*EXT15*	Extensin	1.74	2.71	1.72	1.16
AT3G54590	*EXT2*	Extensin	1.69	3.18	2.03	1.65
AT4G13390*	*EXT18*	Extensin	1.67	2.68	1.57	-
AT5G57530*	*XTH12*	Xyloglucan endotransglucosylase	1.66	2.07	1.49	1.28
AT3G60280*	*UCC3*	Uclacyanin	1.63	2.63	1.57	1.14
AT3G49960		Peroxidase	1.54	2.76	1.77	1.38
AT1G27730	*ZAT10*	Zinc finger protein	1.54	1.13	1.14	2.19
AT1G62440	*LRX2*	Leucine-rich repeat extensin	1.52	1.71	-	-
AT4G29780		Unknown protein	1.50	1.19	1.52	2.54
AT3G54580	*EXT10*	Extensin	1.50	2.82	1.77	1.46
AT1G12040	*LRX1*	Leucine-rich repeat extensin	1.47	2.68	1.64	1.31
AT3G62680*	*PRP3*	Proline-rich protein	1.43	2.48	1.51	1.13
AT5G22410*	*RHS18*	Peroxidase	1.39	2.43	1.75	1.18
AT5G24140	*SQP2*	Squalene monooxygenase	1.36	3.02	2.65	2.01
AT4G22080*	*RHS14*	Pectate lyase	1.35	2.36	1.64	1.17
AT2G37870		Lipid transfer protein	1.34	3.22	3.00	3.21
AT5G67400*	*RHS19*	Peroxidase	1.32	2.60	1.79	1.16
AT3G19430		Late embryogenesis abundant protein	1.30	2.78	2.04	2.17
AT4G25820*	*XTH14*	Xyloglucan endotransglucosylase	1.25	2.54	1.76	1.31
AT3G45140	*LOX2*	Lipoxygenase	1.20	2.86	2.07	2.79
AT4G02270*	*RHS13*	Extensin-like protein	1.17	2.03	1.18	-
AT2G25160	*CYP82F1*	Cytochrome P450	1.17	1.38	1.56	1.33
AT4G40090*	*AGP3*	Arabinogalactan protein	1.15	2.14	1.10	-
AT5G04960		Pectin methylesterase inhibitor	1.15	2.29	1.54	1.06
AT4G11280	*ACS6*	ACC synthase	1.13	1.82	1.91	2.49
AT1G12950	*RHS2*	MATE transporter	1.09	2.50	2.10	1.75
AT4G34530	*CIB1*	Helix-loop-helix transcription factor	1.07	1.24	-	-
AT5G24160	*SQE6*	Squalene monoxygenase	1.07	2.51	1.68	1.87
AT4G34580*	*COW1*	Phosphatidylinositol transfer protein	1.06	1.57	-	-
AT3G60330	*AHA7*	H(+)-ATPase	1.00	2.72	2.03	1.46
AT3G10710*	*RHS12*	Pectinesterase	1.00	2.42	1.62	1.21

Note: The nomenclature of extensins is according to [46]. For some genes, a specific gene name is not assigned, which is left as blank.

(-): The transcripts which are either not detected or their expression level is below Log2 = 1 in RNA-seq experiments.

(*): The genes which are also the putative targets of RSL4.

Together, the results suggest that the transcription of these 37 starvation-induced genes is regulated by ethylene signaling via the modulation of levels of EIN3/EIL1 proteins.

### Functional analysis of the putative root hair-related genes

**Fig 9:**
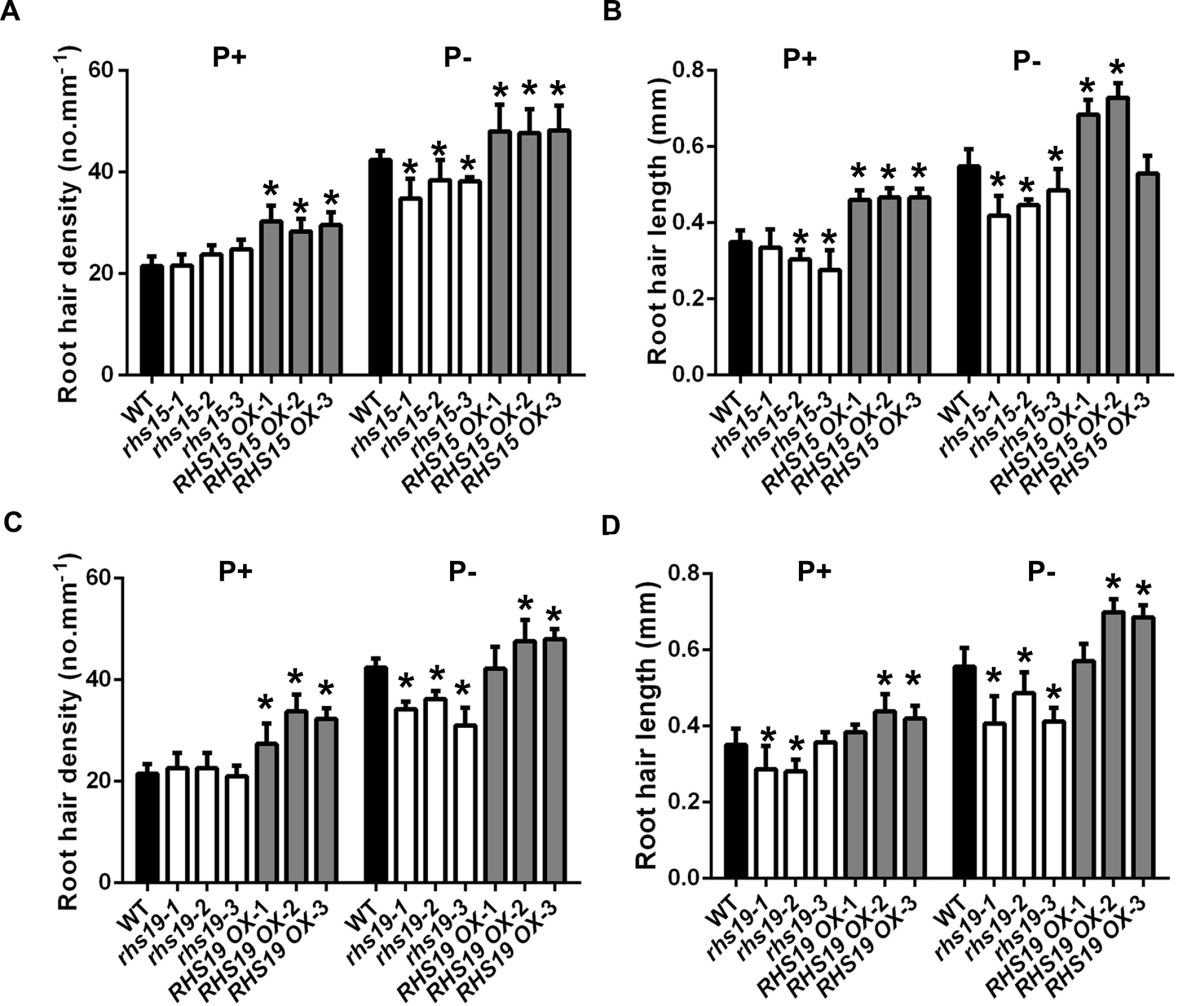
The root hair density and length of the WT, the T-DNA insertion lines, and *RHS15 OX* and *RHS19 OX* lines grown under P+ and P- conditions. Values are the means ± SD of at least 10 7-day-old seedlings. Asterisks indicate a significant difference from the WT (*t*-test, P < 0.05).

To confirm the function of some upregulated genes in *hps5* in root hair development, we examined the phenotypes of the T-DNA insertion lines for 14 genes; we found that the T-DNA lines for six genes (*EXT13*, At2g16586, *UCC3*, *ORA59*, *RHS15*, and *RHS19*) showed some defects in root hair development. Because *RHS15* and *RHS19* are specifically expressed in root hairs [29], we conducted a detailed analysis of the root hair phenotypes for these two genes using their T-DNA insertion lines and overexpressing lines, which were generated using a root hair-specific *EXP7* promoter [47]. Three homozygote SALK lines containing T-DNA insertions in the promoter of *RHS15* (SALK_015573, SALK_015669, and SALK_117952), and three homozygote SALK lines containing T-DNA insertions in the third exon (SALK_093852), the promoter (SALK_010873), and the 3’UTR (SALK_020724) of *RHS19*, respectively, were identified (S16A Fig) by genotyping using the primers listed in S5 Table. qPCR analyses showed that the three SALK lines of *RHS15* were knockdown lines and that the three *RHS19* T-DNA lines were nearly null mutants (S16B and S16C Fig). Under Pi sufficiency, root hair density of all three T-DNA lines for *RHS15* and *RHS19* did not significantly differ from that of the WT; however, root hairs were shorter for two *rhs15* lines and two *rhs19* lines than for the WT (Fig 9). Under Pi deficiency, both root hair density and length of all three T-DNA lines for *RHS15* and *RHS19* were reduced compared to the WT. In the overexpressing lines for these two genes (S16B and S16C Fig), in contrast, root hair density and length tended to be greater than for the WT under both P+ and P- conditions (Fig 9).

### EIN3 directly binds to the promoters of upregulated genes in *hps5*

**Fig 10:**
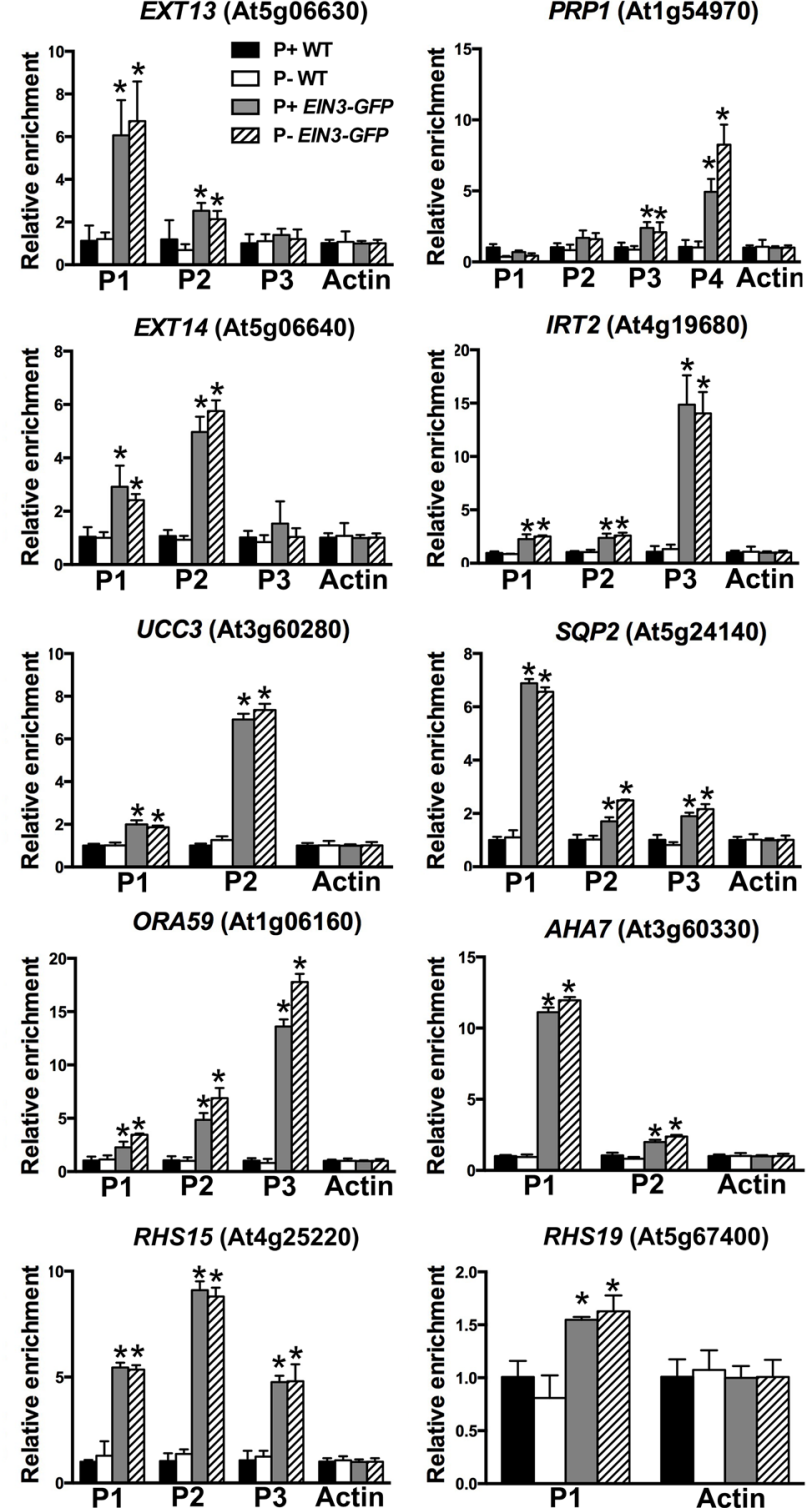
ChIP-qPCR assays of the binding of the EIN3 to the promoters of ten selected genes. Chromatins from the WT and *35S*::*EIN3-GFP* transgenic plants were isolated and immuno-precipitated with GFP-Trap. The levels of enrichment of the precipitated DNA fragments were quantified by qPCR assays. An actin gene fragment was amplified as the internal control. Values are means ± SD of three replicates. Experiments were repeated two times with similar results. Asterisks indicate a significant difference from the WT (*t*-test, P < 0.05).

EIN3/EIL1 bind to DNA with a consensus sequence of A(C/T)G(A/T)A(C/T)CT [48]. We then analyzed the promoter regions of 12 upregulated genes in P+ *hps5* and found that all of them contained 1 to 8 putative EIN3-binding sites (S17 Fig). To determine whether EIN3 can directly bind to these promoters, we performed chromatin immunoprecipitation (ChIP) assays using the transgenic line expressing an *35S*::*EIN3-GFP* fusion gene [26]. The chromatins of 10-day-old seedlings of the WT and *35S*::*EIN3-GFP* line grown under P+ or P- conditions were isolated, cross-linked, and precipitated with anti-GFP antibodies. The DNA fragments that precipitated with EIN3-GFP proteins were analyzed by qPCR. The results showed that PCR-amplified EIN3-binding sites in 10 of the 12 selected genes were enriched in the immuno-precipitated chromatins from the EIN3-GFP plants but not from the WT plants (Fig 10). These results indicated that EIN3 could directly bind to one or more EIN3-binding sites on the promoters of these 10 genes, including *RHS15* and *RHS19*, *in planta*. The binding affinity to EIN3, however, varied among sites. The binding of EIN3 to most of these promoters was not further enhanced by Pi starvation, probably because of the overexpression of *EIN3* as directed by the strong 35S promoter.

**Fig 11:**
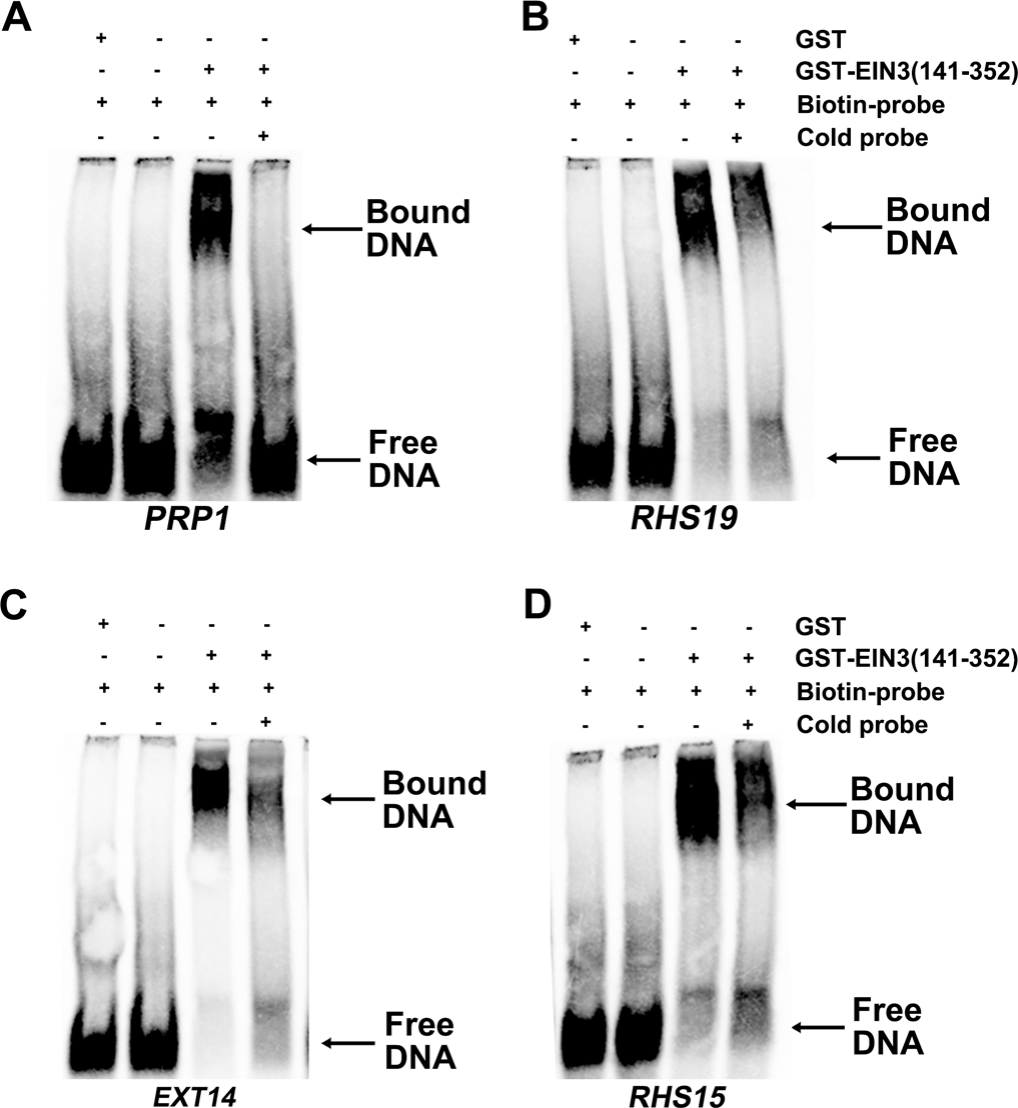
EMSA assays showing the binding of EIN3-GST to the EIN3-binding sites of the promoters of four selected genes. The gene names are indicated at the bottom of each panel. They are *PRP1* (A), *RHS19* (B), *EXT14* (C), and *RHS15* (D). The experiment was performed using 0.1 μg of EIN3-GST or GST proteins. The biotin-labelled probe contained 10 fmol of the EIN3-binding element. The 500 X excess unlabeled probe (cold probe) was used as a competitor.

To provide more evidence that EIN3 can directly bind to the promoter of these genes, we carried out electrophoretic mobility shift assays (EMSA) using recombinant EIN3 proteins produced in *E*. *coli* cells. The recombinant proteins contain the DNA-binding domain of EIN3 (amino acids 141–352). This truncated EIN3 protein was fused with a GST protein to facilitate protein purification. The DNA probes used in EMSA were tandemly repeated tetramers of the putative EIN3-binding elements with 5-bp flanking sequences on each side (The sequences of the DNA probes are listed in S6 Table). The EIN3-binding elements tested in the EMSA assays were selected from promoters of four genes (*PRP1*, *EXT14*, *RHS15*, and *RHS19*) that showed high binding affinity to EIN3 in the ChIP assays. The EMSA results indicated that the recombinant GST-EIN3 proteins, but not GST protein alone, can bind to the biotin-labelled probes. The binding of EIN3-GST proteins to the biotin-labelled probes could be competed out by unlabelled probes, indicating that the binding is sequence-specific (Fig 11).

### EIN3 and RSL4 share some direct targets involved in root hair development

RSL4 (ROOT HAIR DEFECTIVE 6 [RHD6]-LIKE4) is a basic helix-loop-helix transcription factor that is important for root hair development. The loss-of-function mutant of *RSL4* had reduced root hair density and root hair length [49]. In contrast, the *RSL4 OX* lines had increased root hair density and root hair length. Yi et al. [49] further showed that the transcription and protein accumulation of RSL4 are enhanced by Pi starvation and that the induction of root hair growth by Pi starvation is impaired in *rsl4*. Eighty-three putative direct targets of RSL4 were identified by comparative RNA-seq analyses using the WT, *rsl4*, and *RSL4 OX* lines [49]. We compared these 83 genes and 61 upregulated genes in P+ *hps5* and found that 19 genes were in common (Table 1, the genes indicated by asterisks). Among them, the functions of seven genes in root hair development have been experimentally demonstrated. These results suggested that EIN3 and RSL4 might bind to the same promoters of a subset of downstream target genes involved in root hair development.

To determine whether *RSL4* itself is regulated by ethylene signaling, we examined *RSL4* expression in 7-day-old seedlings grown on P+ and P- media in the absence or presence of ACC. The results indicated that the expression of *RSL4* was induced by Pi starvation, which was consistent with [49], but was not induced by ACC (S18 Fig).

## Discussion

As an important adaptive response to Pi deficiency, plants enhance their root hair production, including the increase in both root hair density and root hair length. This response is thought to increase the surface area of roots for Pi uptake and root exudation. Root hair development induced by Pi starvation is mediated by the phytohormone ethylene [12]. Although the ethylene signaling pathway has been well elucidated [14], the molecular mechanism of how ethylene mediates Pi starvation-induced root hair development remains unknown.

In this work, we identified an *Arabidopsis* mutant, *hps5*, that displays enhanced root hair production under both Pi sufficiency and deficiency (Fig 1). *hps5* also has other altered responses to Pi deficiency, and these include increased root-associated APase activity, increased Pi starvation-induced gene expression, and reduced anthocyanin accumulation (S2–S4 Figs). These altered Pi responses are caused by a mutation in the ethylene receptor ERS1 (Fig 2). By analyzing three *Arabidopsis hps* (*h**ypersensitive to* *P**i* *s**tarvation*) mutants (*hps2*, *hps3*, and *hps4*), we previously showed that enhanced ethylene biosynthesis or ethylene signaling makes plant hypersensitive to Pi starvation [50,51,20]. The Pi responses of *hps5* are similar to those of these three *hps* mutants and are consistent with an enhancement of ethylene signaling. This inference is further supported by the enhanced apical hook and increased expression of ethylene-responsive genes in *hps5* seedlings, and by the retarded growth of plants overexpressing the mutated *ERS1* gene (Figs 3–5). On the other hand, Pi deficiency has been shown to increase plant sensitivity to ethylene (for a review, see [13]). For example, the effects of ethylene on the induction of the Pi transporter gene *Pht1;4* [50] and root hair development [12] were much stronger under Pi deficiency than under Pi sufficiency, indicating a cross-talk between ethylene and phosphorus availability. Enhanced ethylene biosynthesis or signaling is known to induce the accumulation of EIN3 protein [26]. The results of the current study show that ethylene induces greater EIN3 accumulation when plants are exposed to Pi starvation (Fig 6), suggesting that Pi starvation increases the stability of EIN3. Therefore, EIN3 may act as the convergent point for the cross-talk between ethylene and low-Pi signal. That Pi deficiency increases the accumulation of EIN3 protein may also be the molecular basis for how Pi deficiency enhances plant sensitivity to ethylene.

Phenotypic and transcriptomic analyses of *hps5* provided us with a good opportunity to identify the downstream targets of the ethylene signaling involved in Pi starvation-induced root hair development (Fig 8). Ethylene has multiple effects on plant growth and development, including the inhibition of root growth and the promotion of root hair production, leaf and flower senescence, and fruit ripening [52]. Because the morphological changes in *hps5* were evident only for roots at the seedling stage, the root seems to be the most sensitive organ in response to the ethylene signal. Thus, a transcriptomic analysis of *hps5* may help identify the target genes of ethylene signaling involved in root development, including root hair formation.

**Fig 12:**
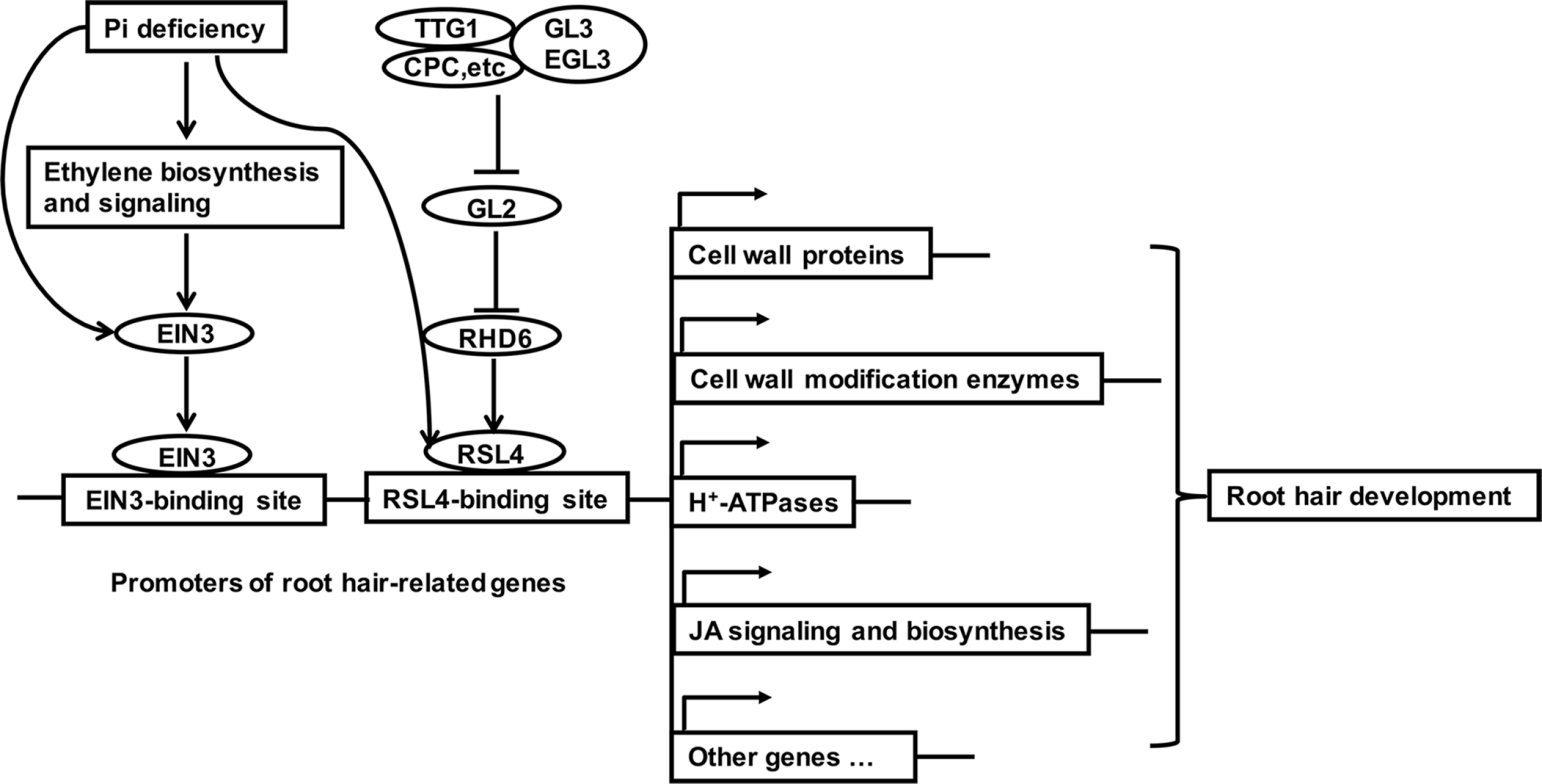
A model showing how ethylene enhances the expression of RLS4-targeted genes to promote root hair development. Above the promoter region, arrows indicate promotion, and perpendicular lines indicate inhibition.

Root hair formation involves three steps, i.e., cell fate specification, hair initiation, and hair elongation. An elegant regulatory pathway regulating hair cell specification has been established in *Arabidopsis* [35,53,54]. GL2 is a negative transcriptional regulator of root hair formation. In the hair cells, an upstream complex consisting of TTG1, GL3, EGL3, CPC, and other proteins suppresses the expression of *GL2* and thus releases the expression of its target genes, including a basic helix-loop-helix transcription factor RHD6 and its several homolog RSLs (RHD6-LIKEs) [53]. RSL2 and RSL4 are also the immediate targets of RHD6 [49]. These transcription factors then turn on the expression of a battery of downstream genes that directly participate in the cellular processes involved in root hair initiation and elongation (also see the diagram in Fig 12).

Root hair initiation and subsequent elongation involves polarized cell growth or tip growth that depends on a variety of highly coordinated cellular activities [55,56]. These activities involve the action of cell wall proteins, peroxidases, xyloglucan endotransglucosylases, H+-ATPases, and pectin modification enzymes. In *Arabidopsis*, these cell wall proteins and cell wall modification enzymes are encoded by multigene families. To provide a precise mechanism for the morphogenetic control of root hair development, plants must selectively activate a subset of these genes in root hair cells. However, which specific isoforms of the proteins encoded by these genes are involved in root hair development remain largely unknown. Among the 61 genes that are upregulated in *hps5* relative to the WT under Pi-sufficient conditions, 13 genes had been demonstrated to be involved in root hair development before this work. The co-upregulation of other genes in *hps5*, which have similar biochemical functions or root hair-specific expression patterns as those 13 genes, suggested that they might also participate in root hair development. These other genes include five *RHS* genes (RHS12, a pectinesterase; RHS13, an extensin-like protein; RHS14, a pectate lyase; RHS15, a glycerol-3-phosphate permease; and RHS19, a peroxidase), a xyloglucan endotransglucosylase (XTH12), two other peroxidases (PER13a and At3g49960), nine cell wall proteins (PRP1, PRP3, FLA6, AGP3, EXT2, EXT10, EXT12, EXT13, and EXT15), and one pectin methytransferase inhibitor. Another interesting group of upregulated genes in *hps5* encode five proteins involved in JA biosynthesis and signaling. JA biosynthesis is essential for ethylene-induced root hair formation [57]. Thus, ethylene might regulate root hair development also by altering JA biosynthesis and signaling through the induction of these five genes. The similar co-expression patterns of these genes have also been reported in two other independent studies [32,35]. When we examined the root hair phenotypes of the T-DNA insertion lines for 14 putative root hair-related genes and overexpressing lines of RHS15 and RHS19 (Fig 9), we found that the T-DNA lines for six genes had defects in root hair development. This further supported our inference that these co-expressed genes might also be involved in root hair growth. The lack of root hair phenotypes of the T-DNA lines for the other eight genes might be due to the functional redundancy of genes belonging to the same family or to the involvement of those genes in other aspects of root development, such as primary and lateral root development. In summary, the analyses of the transcriptomic data of *hps5* allowed us to identify the genes encoding the specific isoforms of the cell wall proteins and cell wall modification enzymes involved in the root hair development, including those that have been demonstrated before and new genes discovered in this research.

Because both *hps5* and Pi-deficient plants accumulate high levels of EIN3 protein (Fig 6), we wondered whether EIN3 and its closest homolog EIL1 are directly involved in Pi starvation-induced root hair development. Our RNA-seq analyses of *ein3* and *ein3eil1* mutants indicate that, among the 61 upregulated genes in *hps5* relative to the WT, 43 are also induced by Pi starvation. The induction of 37 of these 43 genes by Pi starvation is partially or completely suppressed in *ein3* and *ein3eil1* mutants (Fig 8). In contrast, their expression is greatly elevated in the *EIN3 OX* lines (based on the analysis of eight randomly selected genes). These promoter of these eight genes all contain putative EIN3-binding elements (S17 Fig). Moreover, our ChIP and EMSA assays further demonstrated that EIN3 can bind to the promoter of these genes (Figs 10 and 11). Together, these results provide compelling evidence that these genes are the direct targets of EIN3.

RSL4 is a key positive regulator of root hair development [49] and acts downstream of GL2 and RHD6. Eighty-three putative direct targets of RSL4 have been identified, including those that function in cell wall modifications. Interestingly, we found that among these 83 putative targets, 19 might also be the direct targets of EIN3 (Table 1). These results indicate that EIN3 and RSL4 bind to the promoters of a subset of the same target genes involved in cell wall modifications. RSL2, a close relative of RSL4, also acts downstream of RHD6 in regulating root hair development [49]. We speculate that EIN3 may also share some targets with RSL2.

In Fig 12, we describe a working model for how ethylene mediates Pi starvation-induced root hair development. We propose that the level of ethylene in root cells is low under normal growth conditions. A group of key transcription factors, including RSL4 and its close relatives (RSL1, RSL2, LRL1, and LRL2) [53], trigger basal level transcription of their target genes to promote root hair initiation and elongation by regulating a variety of cellular activities, such as cell wall modifications (Fig 12). Pi starvation increases the levels of EIN3 proteins in root cells through both enhanced ethylene biosynthesis and protein stability of EIN3. These EIN3 proteins can bypass RHD6 and directly bind to the promoters of the genes targeted by RSL4, RSL2, and probably other transcription factors. At same time, the level of RSL4 proteins is also enhanced by Pi starvation. Thus, elevated levels of EIN3 and RSL4 increase the transcription of these genes, resulting in enhanced root hair production. Ethylene has also been reported to mediate root hair growth induced by other stresses, such as iron and boron deficiencies [58,59], bacterial infection [60], mechanical impedance [61], local water stress [62], and elevated levels of carbon monoxide [63]. Therefore, our working model may provide a general mechanism of how ethylene mediates root hair responses to other stress signals as well.

Our working model is also consistent with previous observations about the effect of ethylene on root hair development under normal growth conditions. Under such conditions, the blocking of ethylene signaling by either an inhibitor of ethylene perception or by mutations in some key components of the ethylene signaling pathway has only a minor effect on root hair initiation and elongation ([12,17,62] and this study). Root hair density and root hair length can be dramatically increased, however, by exogenously applied ACC, by mutations resulting in constitutive ethylene responses (the *ctr1* mutant) or ethylene overproduction (the *eto1* mutant), or by overexpression of *EIN3* [12,15,16,62]. In these plants, the increase of root hair density can be partly attributed to the ectopically formed root hairs (the root hairs formed in the position of non-hair cells). In addition, ethylene has been shown to act downstream or in separate pathway of RHD6 [17]. According to our working model, this is because the contribution of EIN3 to the transcription of the genes involved in cellular processes related to root hair development is relatively low under normal growth conditions. In ACC-treated plants, in mutants with constitutive ethylene response or ethylene overproduction, and in the *EIN3*-overexpressing line, however, the levels of EIN3 proteins in both the hair cells and non-hair cells are dramatically increased. These EIN3 proteins bypass RHD6 to directly bind to the promoters of the RSL4- and RSL2-targeted genes, resulting in a great increase in root hair density and root hair length, including the formation of root hairs in non-hair cells.

Finally, we note that *hps5* will also be very useful for the study of ethylene receptor function. In *Arabidopsis*, there are five ethylene receptors that negatively regulate ethylene signaling. ETR1 and ERS1 belong to the same subgroup in this family. Because of the functional redundancy among these five receptors, a loss-of-function mutation in one receptor does not cause any phenotype [27]. A gain-of-function point mutation, in contrast, can make plants insensitive to ethylene because of a dominant-negative effect [22, 28]. To date, no mutants with a single point mutation in any ethylene receptors have been reported to have constitutive ethylene responses. Previously, all reported point mutations that cause plants to be insensitive to ethylene occur in the receptor’s transmembrane domains. The mutated proline residue in *hps5*, however, is located immediately after the transmembrane domain III (Fig 2A). Therefore, we believe that the sequence of the cytoplasmic region immediately behind the transmembrane domain III is critical for the function of ERS1. The phenotypic analyses of *hps5* and the transgenic plants expressing the mutated *ERS* gene suggest that this region is involved in the protein-protein interactions between ERS1 and other receptors or in self interactions. Further study of the structural–functional relationship of this conserved proline residue in ERS1 may provide new insights into the function of ethylene receptors.

## Materials and Methods

### Plant materials and growth conditions

All plants used in this study were of the Columbia ecotype background. The SALK T-DNA insertional lines were obtained from the Arabidopsis Biological Resource Center (ABRC). The primers used to confirm the insertion of the T-DNA lines are listed in S5 Table. The Pi-sufficient (P+) medium was half-strength MS medium with 1% (w/v) sucrose and 1.2% (w/v) agar (Sigma catalogue no. A1296). In the Pi-deficient (P-) medium, the 1.25 mM KH_2_PO_4_ in the P+ medium was replaced with 0.65 mM K_2_SO_4_. The *Arabidopsis* seeds were surface sterilized with 20% (v/v) bleach for 15 min. After three washes in sterile-distilled water, seeds were sown on Petri plates containing P+ or P- medium. After the seeds were stratified at 4°C for 2 days, the agar plates were placed vertically in a growth room with a photoperiod of 16 h of light and 8 h of darkness at 22–24°C. The light intensity was 100 μmol m ^-2^s ^-1^.

### Mutant isolation

About 100,000 M_2_ seeds representing 6,000 EMS-mutagenized M_1_ plant lines were used for mutant screening. The EMS-mutagenized lines were generated according to [64]. Seeds were plated on P- medium and were grown in the growth room for 7 d. The root hair phenotypes were visually examined under a stereomicroscope (Olympus SZ61, Japan). The seedlings that had enhanced root hair production compared to the WT were identified as putative mutants and were transferred to soil. The plants were self-pollinated, and the mutant phenotypes were confirmed in the next generation. The mutants were back-crossed to the WT plants twice before they were characterized further.

### Quantitative analysis of APase activity

Root surface-associated and total APase activities were quantified as described by [19].

### Quantitative real-time PCR analysis

Total RNAs were extract from 7-day-old seedlings with the TIANGEN RNAprep pure plant kit. A 2-μg quantity of RNA was reverse transcribed in a 50-μl reaction using M-MLV reverse transcriptase (TaKaRa, Kyoto, Japan) according to the manufacturer’s manual. cDNA was amplified with SYBR Premix Ex Taq (TaKaRa) on the Bio-Rad CFX96 real-time PCR detection system. *ACTIN2* gene expression was used as an internal control, and the relative expression level of each gene was calculated by the 2^–△△Ct^ method. The genes and the primers used to detect their mRNA expression are listed in S7 Table.

### Quantitative analysis of cellular Pi and total P contents

Cellular Pi and total P were quantified as described by [19].

### Analysis of anthocyanin accumulation

For analysis of anthocyanin accumulation, the seeds were sown directly on 1/2 MS agar plates (0.6% agar) with different levels of Pi and were placed horizontally in the growth room. The shoots of the seedlings were photographed at 10 DAG using a camera attached to a stereomicroscope (Olympus SZ61, Japan).

### Genetic mapping of the *HPS5* gene

The mapping population was generated by crossing the mutant *hps5* to a plant of the Landsberg *erecta* ecotype. F_2_ progeny that displayed the dark-blue BCIP staining phenotype were selected, and DNAs from these seedlings were isolated for molecular mapping. A set of simple sequence length polymorphism (SSLP) and cleaved-amplified polymorphic sequence (CAPS) markers was used to map the *HPS5* gene. The sequences and chromosomal positions of the molecular markers are listed in S8 Table.

### Treatment of the ethylene precursor

For the test of triple responses, seeds were sown directly on P+ medium (0.6% agar) containing 1 μM ACC. After the seeds were stratified at 4°C for 3 days, they were placed horizontally in growth room at 22°C in the dark. After 96 h, the seedlings were photographed and the length of hypocotyl was measured. At least 20 seedlings (n> 20) were scored for each measurement.

### Confocal microscopy

For observation of the accumulation of EIN3-GFP proteins, seeds were first germinated on P+ medium (1.2% agar) and grown for 4 days. The seedlings were then transferred to P+ and P- media with or without 10 μM ACC. After 6 days on these media, the roots were excised from the seedlings and were examined with a confocal laser scanning microscope (Zeiss LSM710, Germany). Excitation wavelengths of 488 nm and emission wavelengths of 507 nm were used to visualize GFP signals.

### Generation of transgenic plants

To generate the plant transformation vector 35S::*ERS1* for genetic complementation, the genomic sequence of the *ERS1* gene was amplified from genomic DNA of WT *Arabidopsis* plants using primers 5’-AGAACACGGGGGACTCTAGAGGATCCATTGGTTTCTTCTTTATCACAC-3’ and 5’-TTGAACGATCGGGGAAATTCGAGCTCGAATCTGCCACAACCACA-3’.

The *ERS1* genomic DNA was cloned into the site after the *CaMV 35S* promoter of the plant expression vector pZH01, which carries a hygromycin-resistant gene. To generate the vector *ERS1*::*ERS1*, the promoter and genomic DNA sequences of *AtERS1* were amplified from WT *Arabidopsis* plants using primers 5'- TCTAGAGGCTAAGAAGTCCGAGAGTA -3' and 5'-CCCGGGGAATCTGCCACAACCACA-3'. The PCR products were cloned into the vector pBI101, which carries a kanamycin-resistant gene. For the vector *35S*:: *mERS1*, genomic sequences of mutated *ERS1* were amplified from the *hps5* mutant using the same primers that were used for vector *35S*::*ERS1*. These constructs were then transformed into the *hps5* mutant plants (*35S*::*ERS1* and *ERS1*::*ERS1*) and WT plants (*35S*:: *mERS1*) by the floral dip method.

To generate the vector *EXPA7*::*RHS15* and *EXPA7*::*RHS19*, the *CaMV 35S* promoter of the plant expression vector pZH01 was replaced by the *EXPA7* promoter sequences. The genomic DNA sequences of *RHS15* and *RHS15* were amplified from WT *Arabidopsis* plants using primers of RHS15-F 5'- AAGAGGCTAGAATGGGATCC CACCATTGTAGTTGAAGAAC -3', RHS15-R 5'- CGATCGGGGAAATTCGAGCTCAGGTAATCTTATTCTCATTC -3', RHS19-F 5'- AAGAGGCTAGAATGGGATCC ACCATTCCTGTAAACACAAA -3', and RHS19-R 5'- GATCGGGGAAATTCGAGCTCGGATTTATTAAACAAGTTTA -3'. The PCR products were cloned into the modified pZH01 vector.

For all of the constructions, the PCR-amplified gene fragments were directly ligated to the plant transformation vector as described in [65].

### Protein extraction and western blots

The 7-d-old Seedlings were ground to fine powders in liquid nitrogen. One volume of ice-cold extraction buffer (0.1 M K-acetate, 20 mM CaCl_2_, 2 mM EDTA, 0.1 mM phenylmethylsulfonyl fluoride, 20% glycerol (v/v), pH 5.4) was added to the powders. Samples were gently agitated on ice for 0.5 h and then centrifuged at 13,000 rpm at 4°C for 15 min. The supernatant was transferred to a fresh tube, and the centrifugation was repeated. About 30 μg of denatured protein was separated on 10% sodium dodecyl sulfate (SDS)-polyacrylamide gels. After electrophoresis, the proteins were transferred to a polyvinylidene difluoride (PVDF) membrane in transfer buffer (25 mM Tris, 43 mM glycine, 20% methanol) and subjected to western blot analysis using anti-EIN3 antibodies as described in [19].

### RNA-seq analyses

The RNA-seq analyses were performed at the Core Sequencing Facility of Tsinghua University. Total RNAs were isolated from 7-d-old seedlings using the RNeasy Plant Mini Kit (Qiagen). Poly(A+) RNAs were purified using the Next Poly(A) mRNA Magnetic Isolation Module (New England BioLabs) and were reversely transcribed into cDNAs using M-MuLV Reverse Transcriptase and Next® mRNA Second Strand Synthesis Module (New England BioLabs). The cDNAs were fragmented and sequencing libraries were prepared by end repairing, “A” tail addition, and adaptor ligation. The libraries were sequenced as 50-mers using HiSeq2500 (Illumina) with standard settings.

The bioinformatics analyses of the sequencing data were essentially carried out as described in [66]. The raw data of Illumina reads are available at the National Center for Biotechnology Information Sequence Read Archive browser (http://ncbi.nlm.nih.gov/sra; accession no. SRP071563).

### ChIP-qPCR assays

ChIP-qPCR assays were performed as described by [67]. A 2-g quantity of 10-d-old *35S*::*EIN3-GFP* seedlings was used for ChIP assays. The chromatins were precipitated using GFP-Trap A beads. The enriched DNA fragments were released by 200 μM NaCl and subjected to qPCR analysis. The primers used for ChIP-qPCR assays are listed in S9 Table.

### EMSA

To construct a plasmid for the expression of recombinant EIN3 protein (amino acids 141 to 352) in *E*. *coli* cells, the corresponding DNA fragment was amplified by PCR using primers 5’-ACTGGATCCAAGGTTAGGTTTGATCGT- 3’ and 5’-ACTCTCGAGTCAGAAGAATTCATAACTTTT- 3’. The PCR-amplified fragment was inserted into *BamH*I and *Xho*I sites of the pGEX-6p-1 vector (Pharmacia). The biotin-labeled probes of the promoter sequences were generated by annealing the biotin-labeled complementary oligonucleotides. The sequences of various DNA probes are listed in S6 Table. Each binding reaction (20 μL) contained 0.1 μg of recombinant protein, 20 fM of labeled DNA probe, and 1 μg of poly(dI-dC) in buffer (25 mM HEPES-potassium hydroxide, pH 7.5, 100 mM KCl, 0.1 mM EDTA, 10% [v/v] glycerol, 1 mM DTT); binding reactions were performed at room temperature for 30 min. The binding reaction products were resolved on 5.5% polyacrylamide gels run in 0.5X Tris-borate-EDTA. The free and protein-bound DNAs were detected using the Light Shift Chemiluminescent EMSA kit (Thermo Scientific) according to the manufacturer’s instructions.

## Supporting Information

S1 Fig Primary root length of 7-day-old WT and *hps5* seedlings (A) and lateral root density of 9-day-old WT and *hps5* seedlings (B).
(JPG)

S2 Fig APase activities of the WT and the *hps5* mutant. (A), APase activity detected by BCIP staining on the root surfaces of 7-day-old WT and *hps5* seedlings grown on P+ and P–media. (B), Quantitative measurement of root-associated APase activity of the seedlings shown in (A). Values represent the mean and SD of three replicates. Means with asterisks are significantly different from the WT (P < 0.05, *t*-test).
(JPG)

S3 Fig Relative expression of eight Pi starvation-induced genes in 7-day-old WT and *hps5* seedlings grown on P+ and P- media as determined by qPCR. The expression level for each gene in the WT under P+ condition was set to 1.0. Values are the means and SD of three biological replicates and represent fold changes normalized to transcript levels of the WT on P+ medium. Means with asterisks are significantly different from the WT (P < 0.05, *t*-test).
(JPG)

S4 Fig Anthocyanin accumulation in the WT and the *hps5* mutant grown on P+, P-, and P- medium supplemented with different amounts of Pi as indicated on the top. The pictures were taken 10 days after seed germination.
(JPG)

S5 Fig Cellular Pi contents (A) and total phosphorus (B) in 9-day-old WT and *hps5* seedlings grown on P+ and P–medium.
(JPG)

S6 Fig The strategy for fine mapping of the *HPS5* gene. The molecular markers used in the fine mapping are shown above the horizontal lines. The numbers below each horizontal line are the AGI coordinates on the chromosome. Distance unit: Mb. The position of the mutation is indicated at the bottom.
(JPG)

S7 Fig Alignment of a part of protein sequences of ERS1 and its orthologs in other plant species. The alignment was generated using CLUSTAL program (Higgins et al. 1996). Identical and similar amino acids among the different plant species are highlighted with black and grey background, respectively. Numbers at the left indicate the positions of amino acid residues. Red box indicates the conserved amino acids which is mutated in *hps5*.
(JPG)

S8 Fig Genetic complementation of the *HPS5* gene in term of root-associated APase activity. (A), BCIP staining of root-associated APase activity in 7-day-old seedlings of WT, *hps5*, and two complementation lines (*ERS1*::*ERS1* and *35S*::*ERS1*) grown on P+ and P- media. (B), A close view of the roots of the seedlings shown in (A).
(JPG)

S9 Fig Morphology of 25-day-old WT and *hps5* plants grown in soil. (JPG)

S10 Fig Relative expression levels of the *ERS1* gene in the 7-day-old seedlings of the WT, *hps5*, and six *35S*::*mERS1* transgenic lines grown under P+ and P- conditions determined by qPCR. (JPG)

S11 Fig Primary root length in 7-day-old seedlings of the WT, *hps5*, and six *35S*::*mERS1* transgenic lines grown under P+ and P- conditions. (JPG)

S12 Fig Root-associated APase activity of various plant lines. (A), BCIP staining of root-associated APase activity of 7-day-old seedlings of the WT, *hps5*, and six *35S*::*mERS1* transgenic lines grown under P+ and P- conditions. (B), A close view of the root-associated APase activity of the seedlings shown in (A).
(JPG)

S13 Fig Relative expression level of *EIN3* in roots of 7-day-old *Arabidopsis* seedlings grown under various conditions as determined by qPCR. (A), WT and *hps5* seedlings grown on P+ and P- medium; (B) WT seedlings grown on P+ and P- medium in the absence and presence of 10 μM ACC. The expression level in the WT under the P+ condition was set to 1.0. Values are the means and SD of three biological replicates and represent fold-changes normalized to transcript levels of the WT on P+ medium. *t*-tests were performed to test the significance.
(JPG)

S14 Fig Relative expression of six selected genes in 7-day-old P+, P- WT, and P+ *hps5* seedlings as determined by qPCR. (JPG)

S15 Fig Relative expression of six selected genes in 7-day-old WT seedlings grown on P+ and P- media and *ein3* and *ein3eil1* seedlings grown on P- medium determined by qPCR. (JPG)

S16 Fig Identification of knock-out mutants and overexpression plants for *RHS15* and *RHS19*. (A) Diagrams showing the positions of the T-DNA in the *RHS15* and *RHS19* gene. Black box: exons; grey box: 5’ and 3’ UTRs; thin line: introns; thick line: flanking sequences surrounding the gene. Relative expression of *RHS15* (B) and *RHS19* (C) in the 7-day-old seedlings of their corresponding T-DNA insertion lines and overexpressing lines as determined by qPCR. The name of SALK lines for each allele of *rhs15* and *rhs19* are as follows. *rhs15-1*: SALK_015573; *rhs15-2*: SALK_015669; *rhs15-3*: SALK_117952. *rhs19-1*: SALK_093852; *rhs19-2*: SALK_010873; *rhs19-3*: SALK_020724.
(JPG)

S17 Fig Schematic diagrams showing the relative positions of the putative EIN3-binding sites (black boxes) in the promoters of 12 upregulated genes in P+ *hps5*. The 1.5 kb upstream sequences are shown, and the translational start sites (ATG) are shown at position +1. Arrows indicates the primers used for ChIP assays. The asterisks indicated the binding sites that is bound by EIN3 proteins.
(JPG)

S18 Fig Relative expression of *RSL4* in 7-day-old WT seedlings grown on P+ and P- medium in the absence or presence of 10 μM ACC determined by qPCR. (JPG)

S1 Table The up- and down-regulated transcripts in P- WT compared to P+ WT. (XLSX)

S2 Table The up- and down-regulated transcripts in P+ *hps5* compared to P+ WT. (XLSX)

S3 Table The downregulated transcripts in P- *ein3* compared to P- WT. (XLSX)

S4 Table The downregulated transcripts in P- *ein3eil1* compared to P- WT. (XLSX)

S5 Table The sequences of the primers used for genotyping of the T-DNA lines. (XLSX)

S6 Table The sequences of the DNA probes used in EMSA. (XLSX)

S7 Table The sequences of the primers used for qPCR analyses. (XLSX)

S8 Table The sequences of the molecular markers used for molecular mapping. (XLSX)

S9 Table The sequences of the primers used for ChIP-qPCR analyses. (XLSX)
