# Supplementary figures and images for: The Molecular Mechanism of Ethylene-Mediated Root Hair Development Induced by Phosphate Starvation
Source: PLoS Genet. 2016 Jul 18;12(7):e1006194. doi: 10.1371/journal.pgen.1006194 (PMC4948871; doi:10.1371/journal.pgen.1006194)

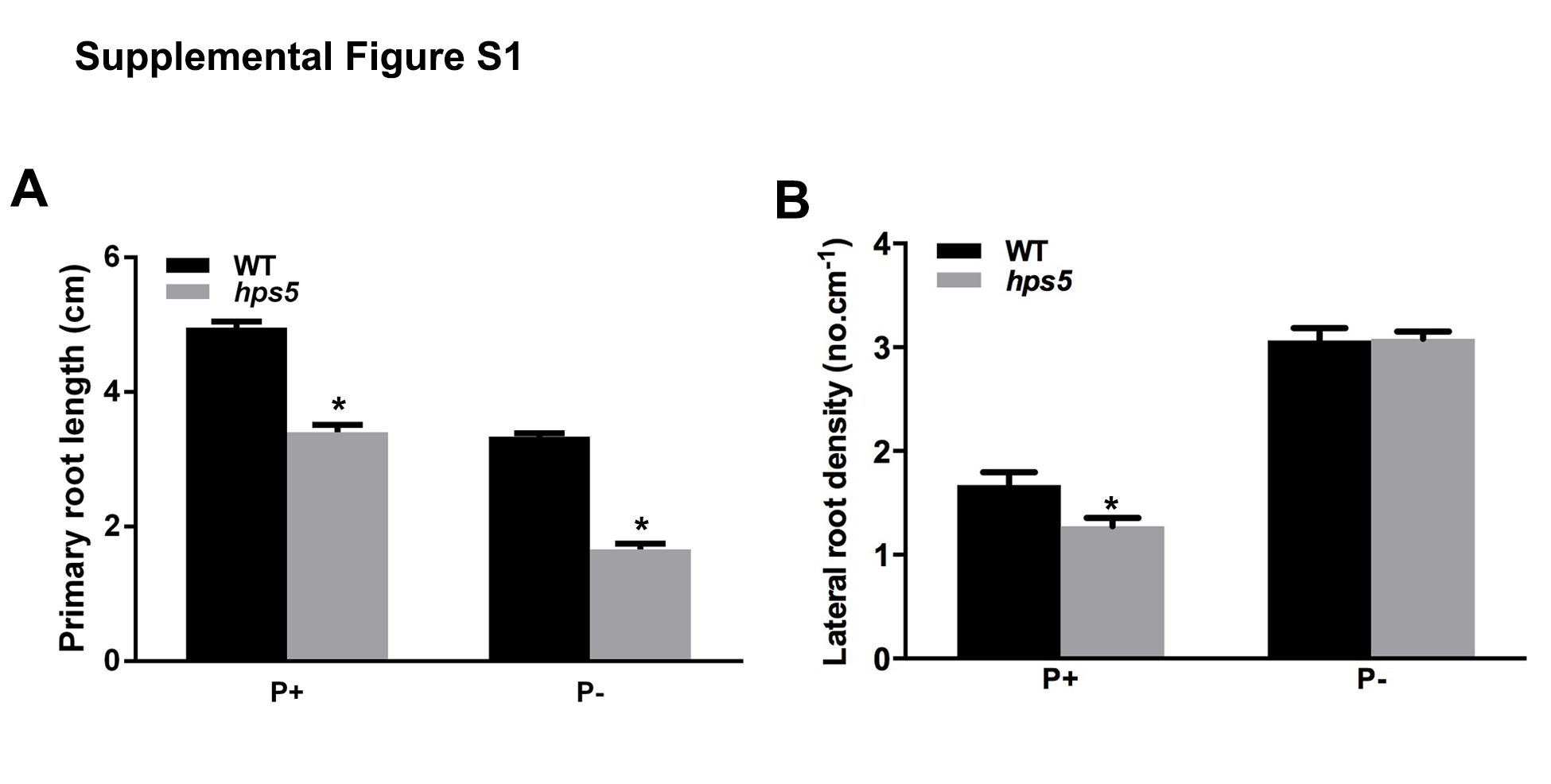

Supplement: S1 Fig — Primary root length of 7-day-old WT and hps5 seedlings (A) and lateral root density of 9-day-old WT and hps5 seedlings (B). (JPG) [file pgen.1006194.s001.jpg]

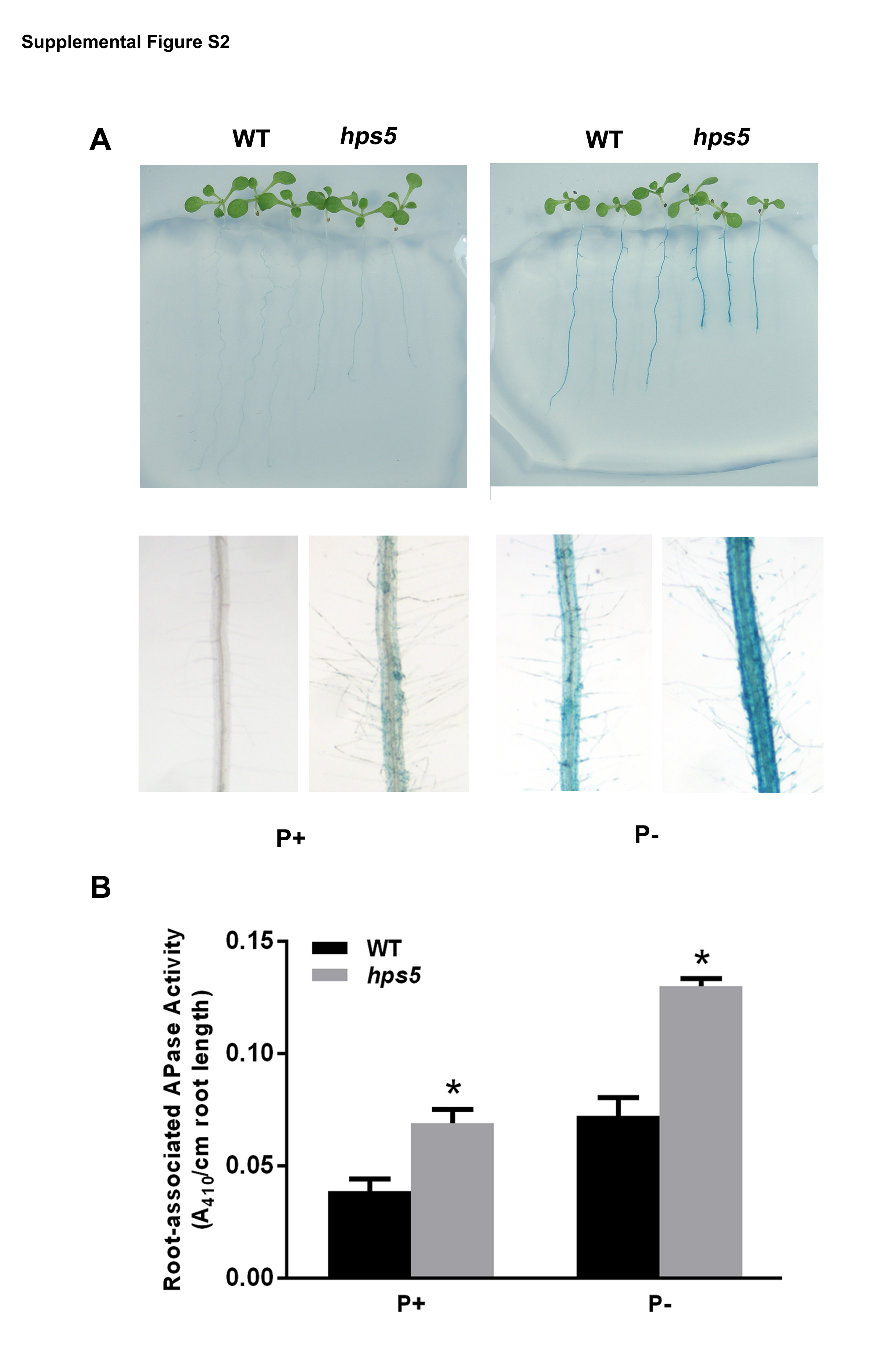

Supplement: S2 Fig — (A), APase activity detected by BCIP staining on the root surfaces of 7-day-old WT and hps5 seedlings grown on P+ and P–media. (B), Quantitative measurement of root-associated APase activity of the seedlings shown in (A). Values represent the mean and SD of three replicates. Means with asterisks are significantly different from the WT (P < 0.05, t-test). (JPG) [file pgen.1006194.s002.jpg]

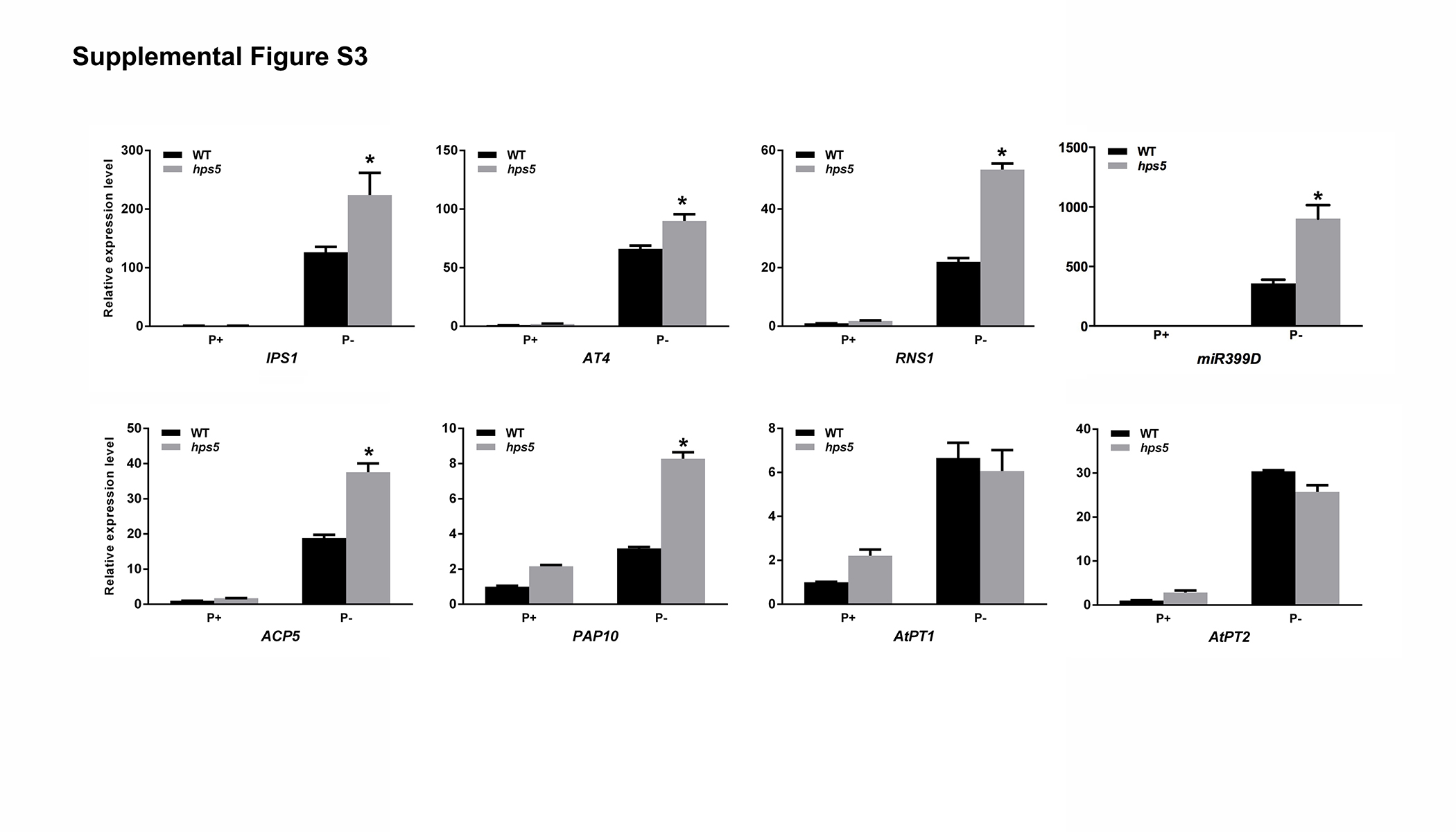

Supplement: S3 Fig — The expression level for each gene in the WT under P+ condition was set to 1.0. Values are the means and SD of three biological replicates and represent fold changes normalized to transcript levels of the WT on P+ medium. Means with asterisks are significantly different from the WT (P < 0.05, t-test). (JPG) [file pgen.1006194.s003.jpg]

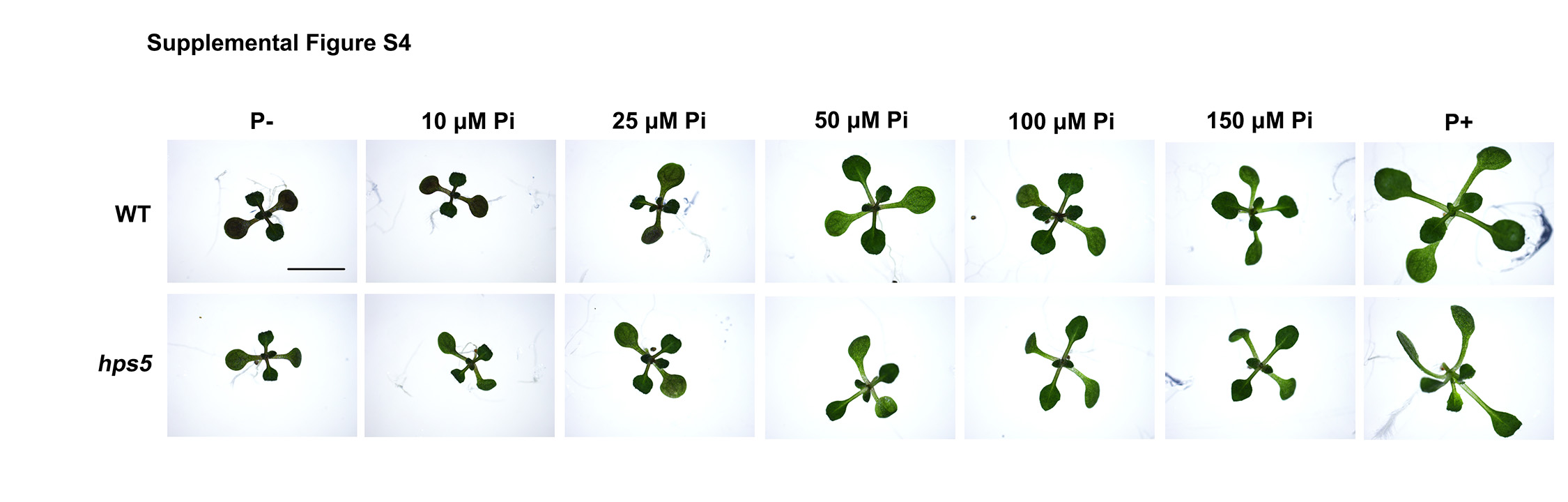

Supplement: S4 Fig — The pictures were taken 10 days after seed germination. (JPG) [file pgen.1006194.s004.jpg]

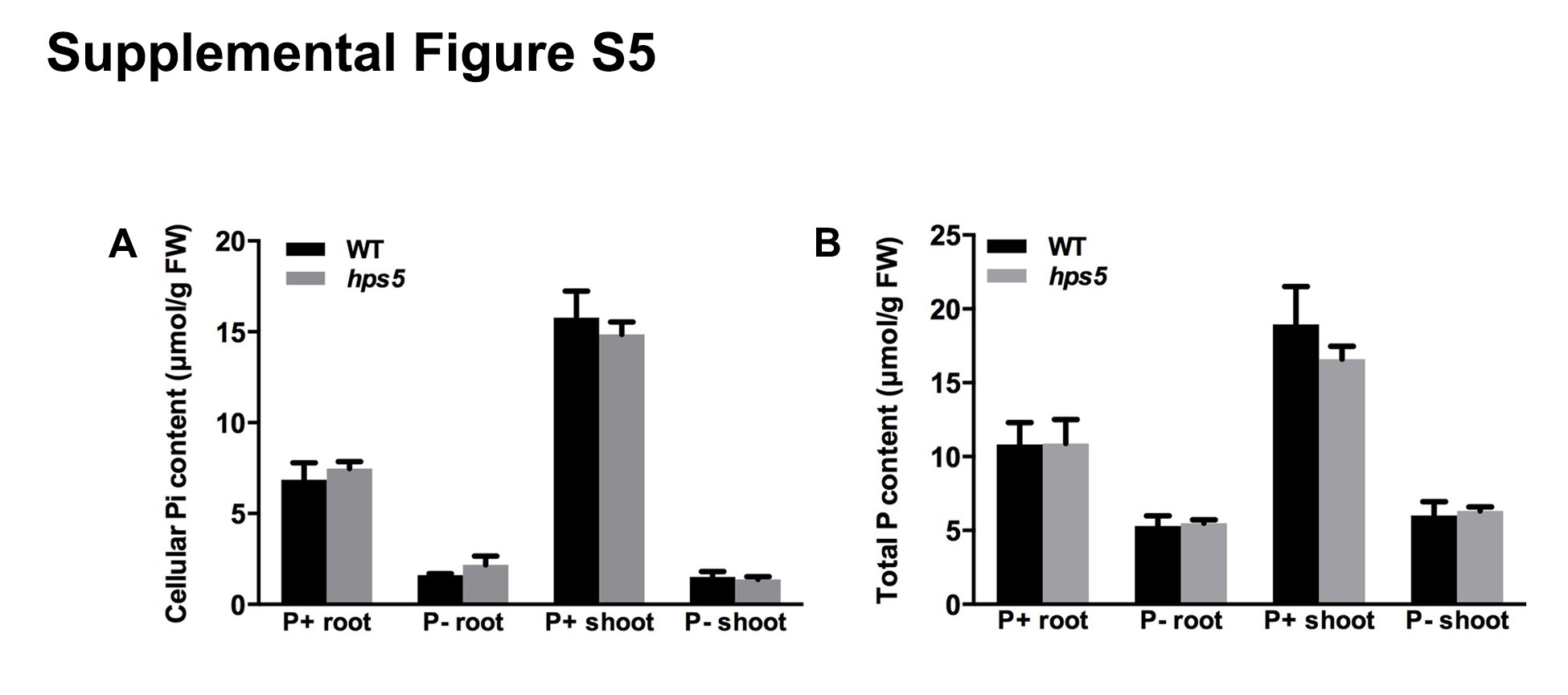

Supplement: S5 Fig — Cellular Pi contents (A) and total phosphorus (B) in 9-day-old WT and hps5 seedlings grown on P+ and P–medium. (JPG) [file pgen.1006194.s005.jpg]

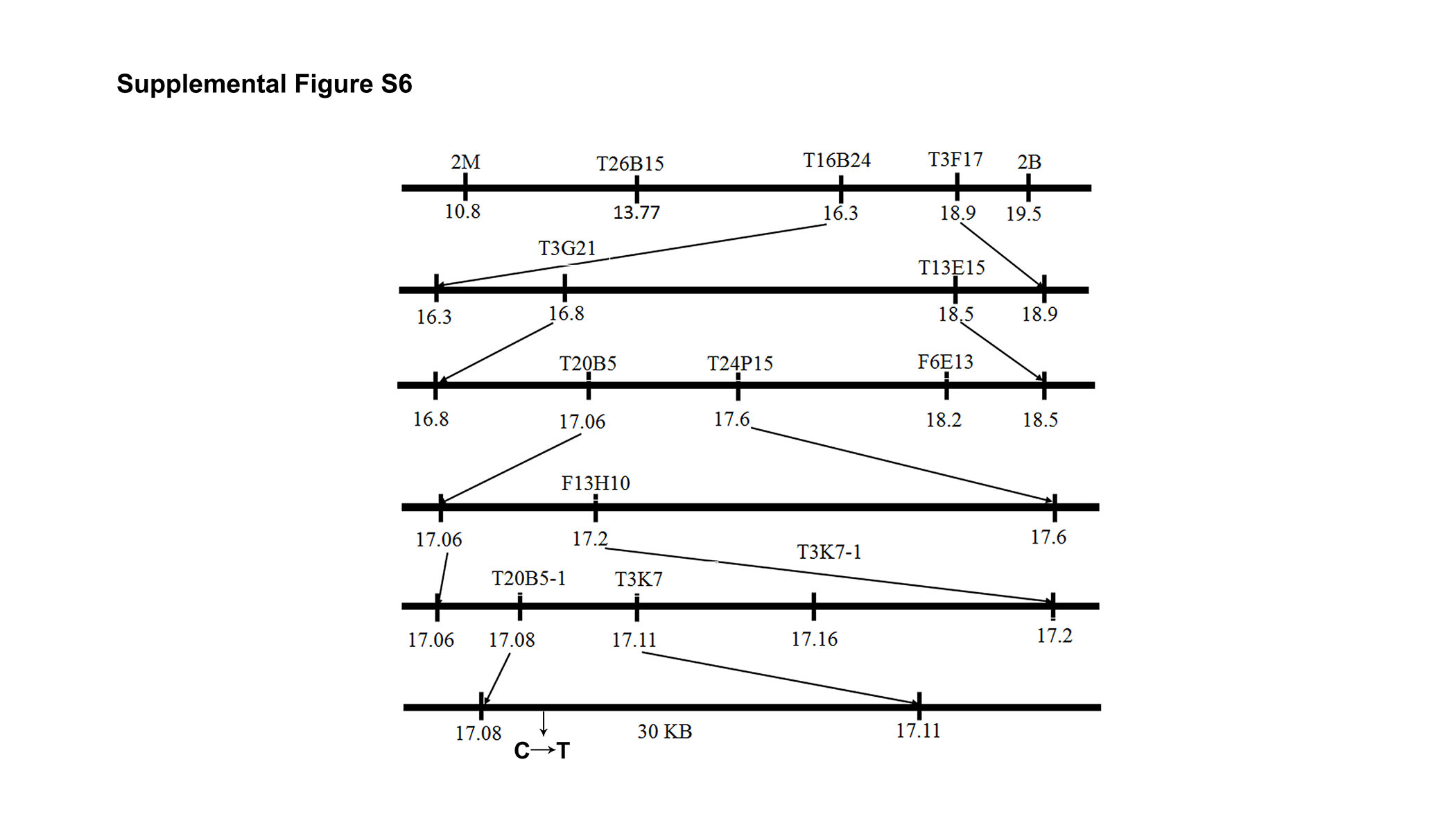

Supplement: S6 Fig — The molecular markers used in the fine mapping are shown above the horizontal lines. The numbers below each horizontal line are the AGI coordinates on the chromosome. Distance unit: Mb. The position of the mutation is indicated at the bottom. (JPG) [file pgen.1006194.s006.jpg]

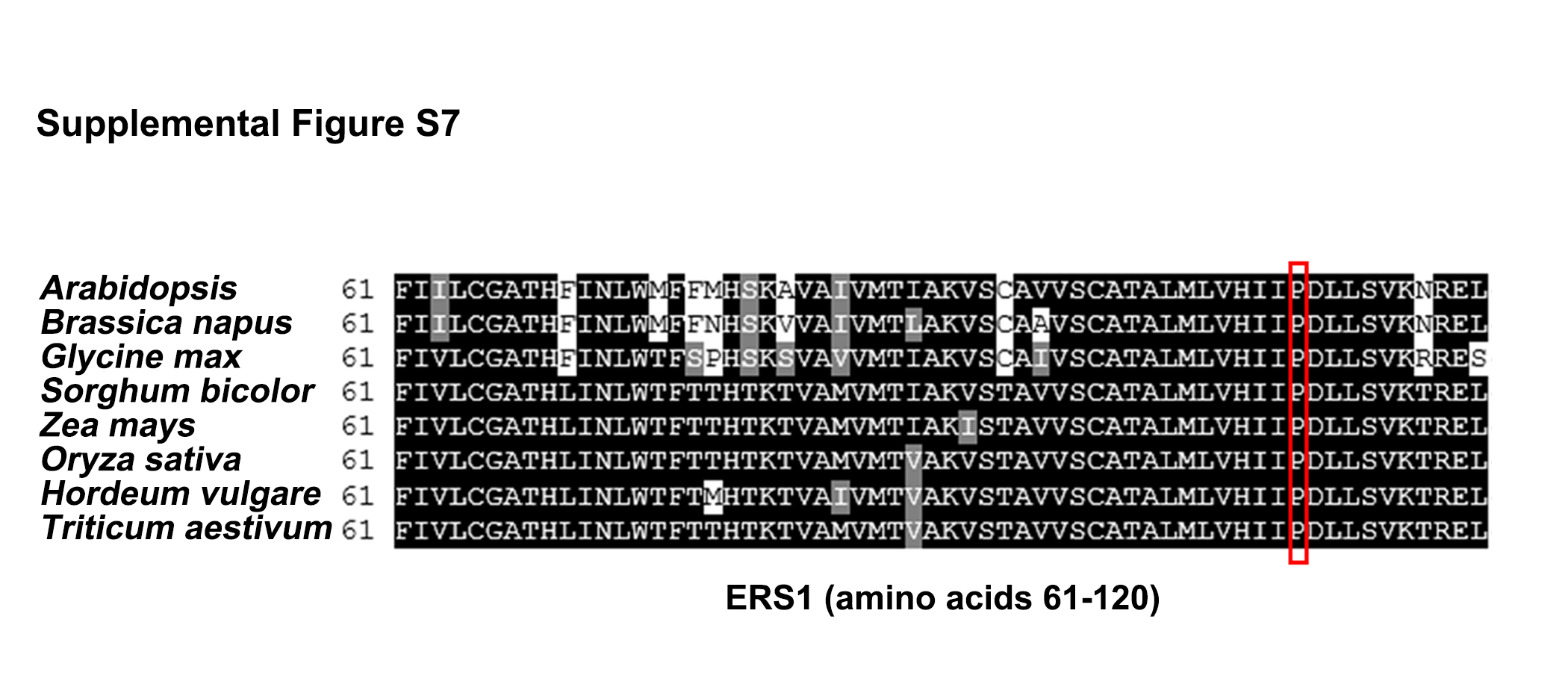

Supplement: S7 Fig — The alignment was generated using CLUSTAL program (Higgins et al. 1996). Identical and similar amino acids among the different plant species are highlighted with black and grey background, respectively. Numbers at the left indicate the positions of amino acid residues. Red box indicates the conserved amino acids which is mutated in hps5. (JPG) [file pgen.1006194.s007.jpg]

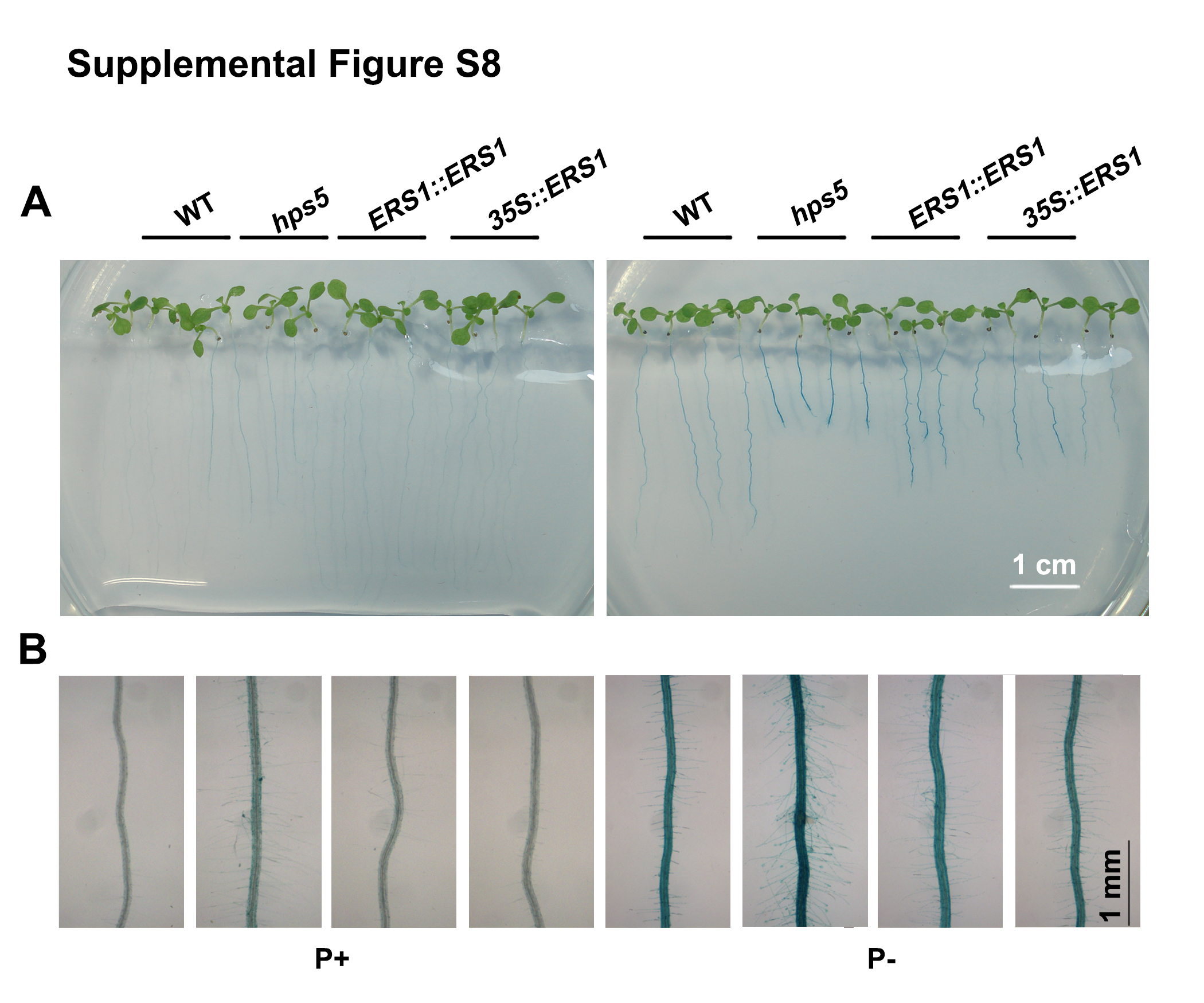

Supplement: S8 Fig — (A), BCIP staining of root-associated APase activity in 7-day-old seedlings of WT, hps5, and two complementation lines (ERS1::ERS1 and 35S::ERS1) grown on P+ and P- media. (B), A close view of the roots of the seedlings shown in (A). (JPG) [file pgen.1006194.s008.jpg]

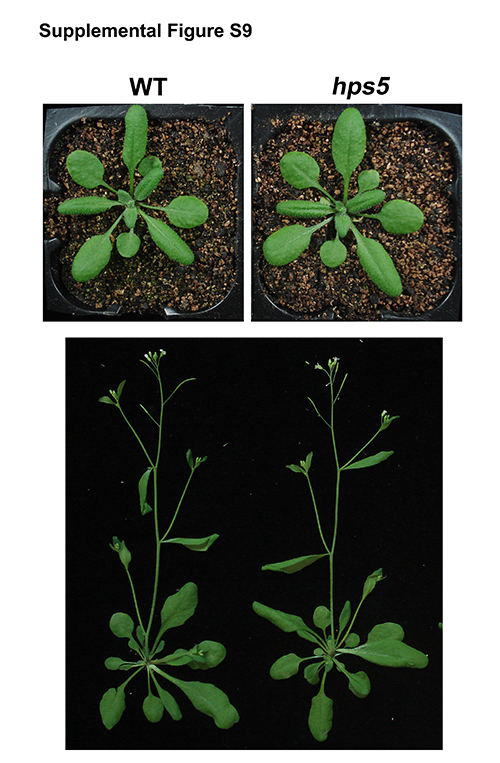

Supplement: S9 Fig — (JPG) [file pgen.1006194.s009.jpg]

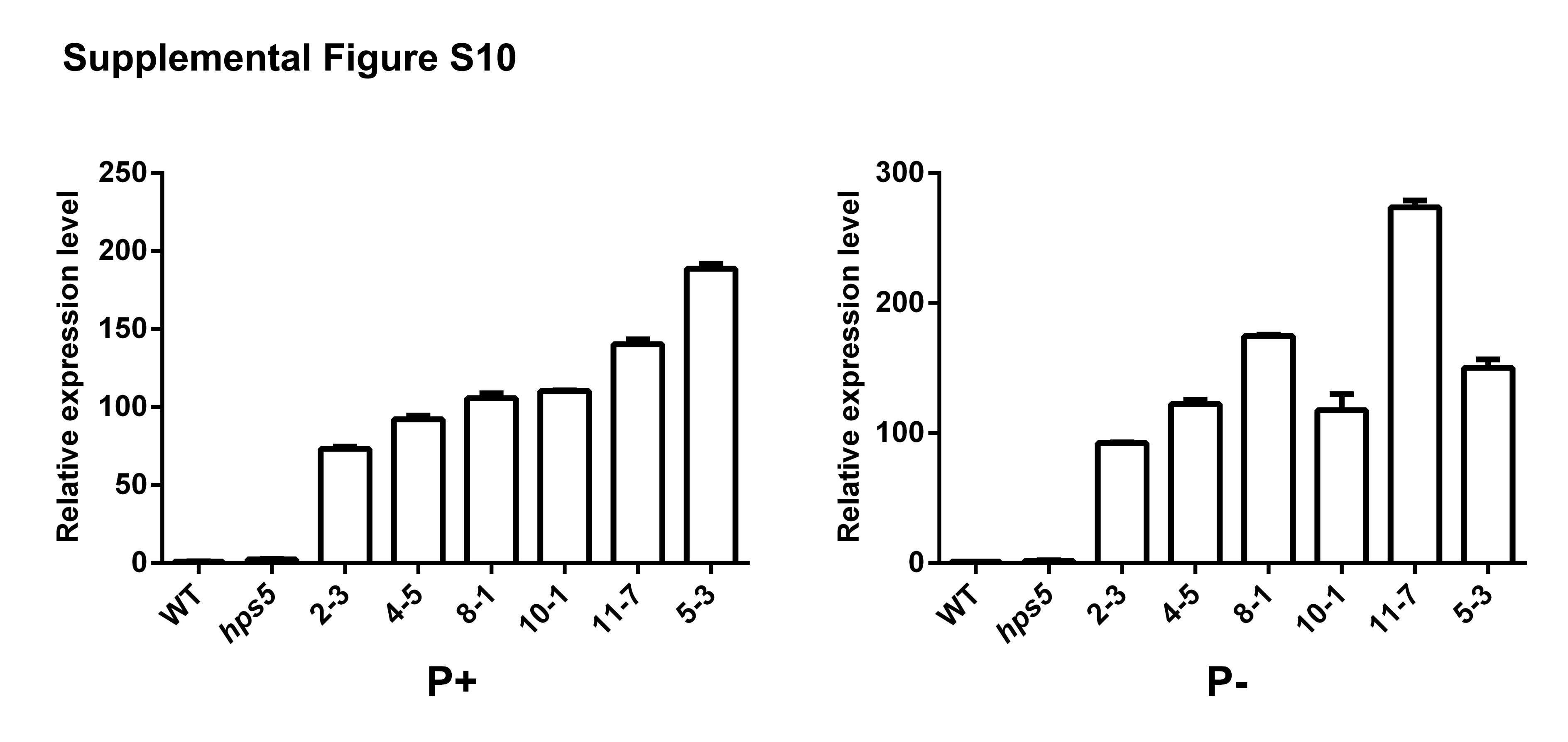

Supplement: S10 Fig — (JPG) [file pgen.1006194.s010.jpg]

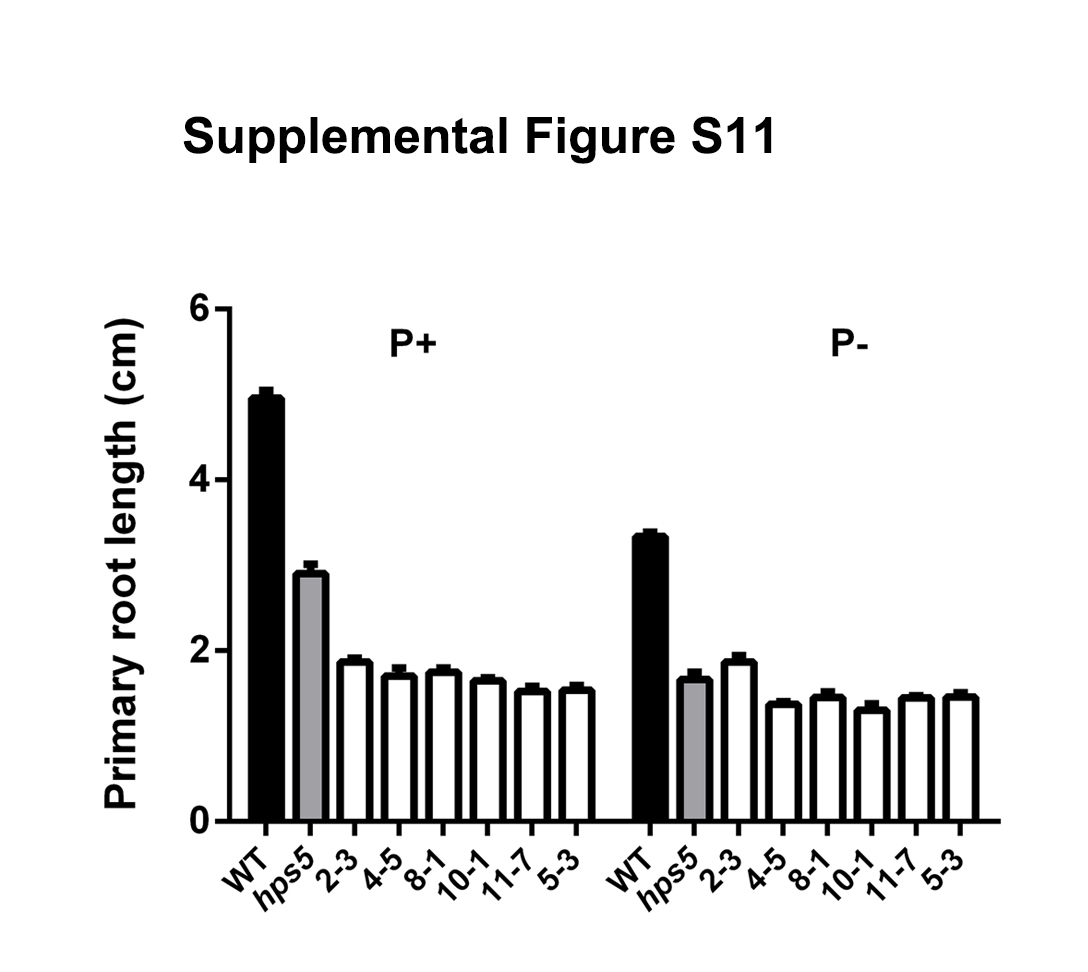

Supplement: S11 Fig — (JPG) [file pgen.1006194.s011.jpg]

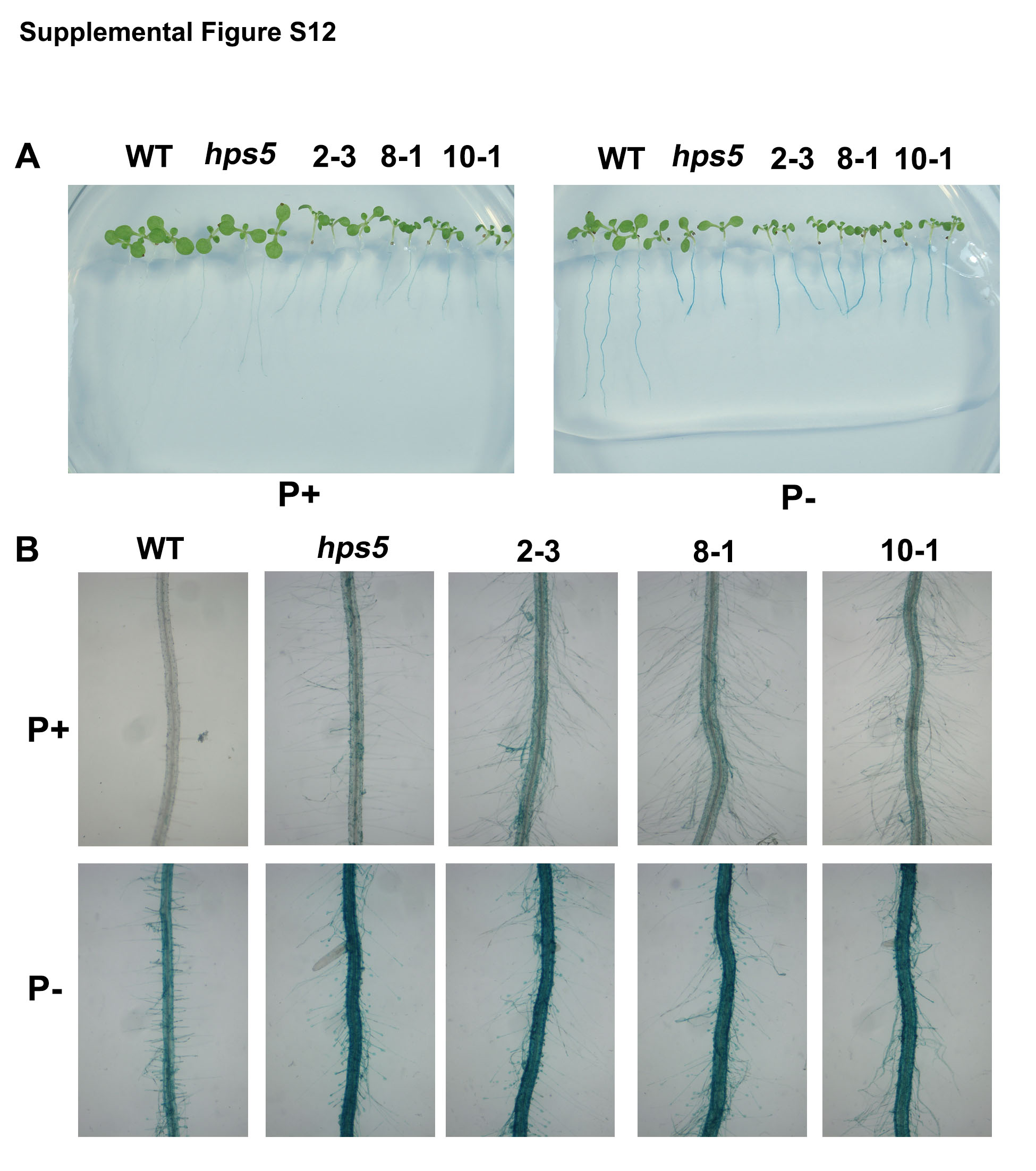

Supplement: S12 Fig — (A), BCIP staining of root-associated APase activity of 7-day-old seedlings of the WT, hps5, and six 35S::mERS1 transgenic lines grown under P+ and P- conditions. (B), A close view of the root-associated APase activity of the seedlings shown in (A). (JPG) [file pgen.1006194.s012.jpg]

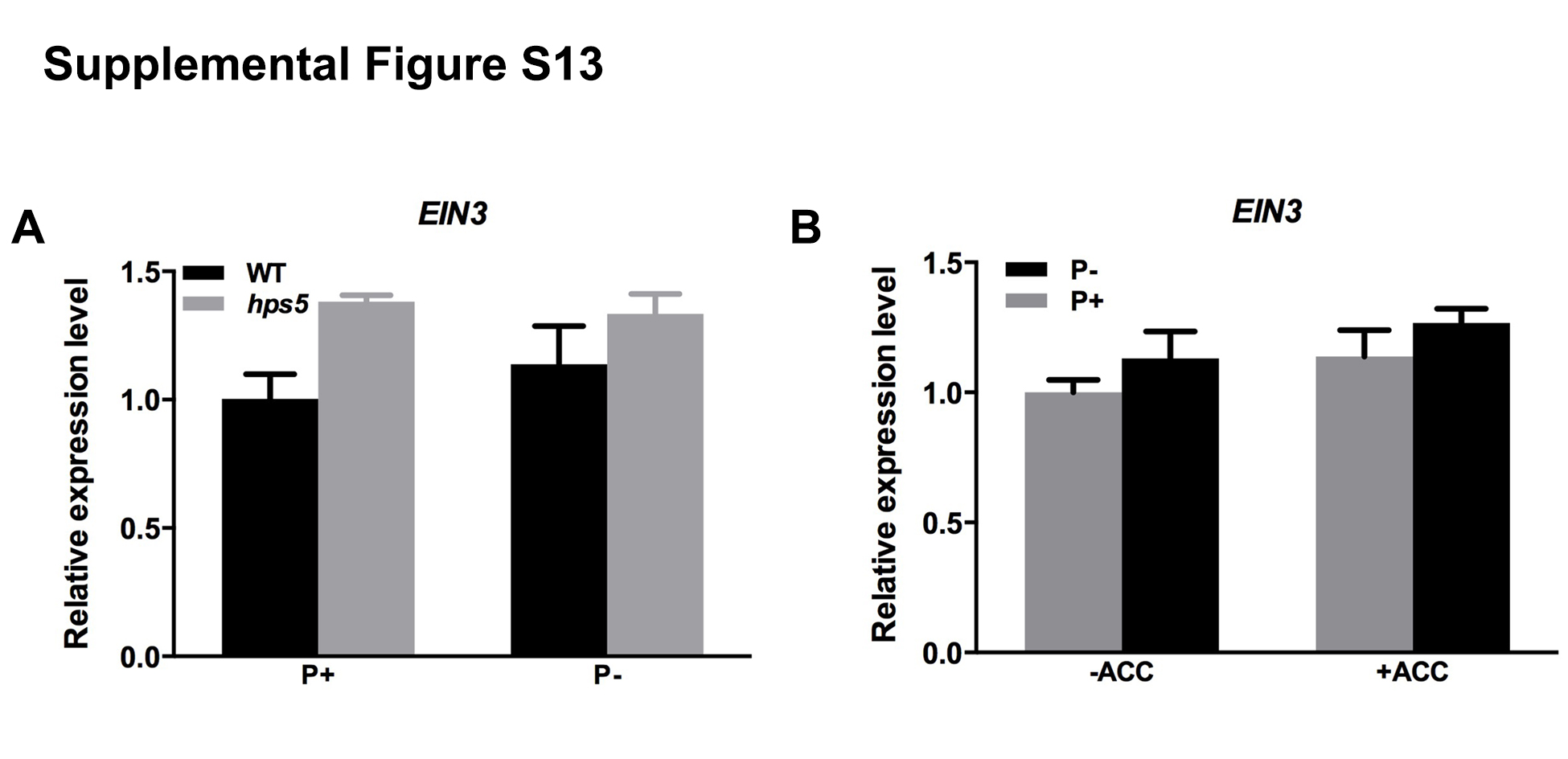

Supplement: S13 Fig — (A), WT and hps5 seedlings grown on P+ and P- medium; (B) WT seedlings grown on P+ and P- medium in the absence and presence of 10 μM ACC. The expression level in the WT under the P+ condition was set to 1.0. Values are the means and SD of three biological replicates and represent fold-changes normalized to transcript levels of the WT on P+ medium. t-tests were performed to test the significance. (JPG) [file pgen.1006194.s013.jpg]

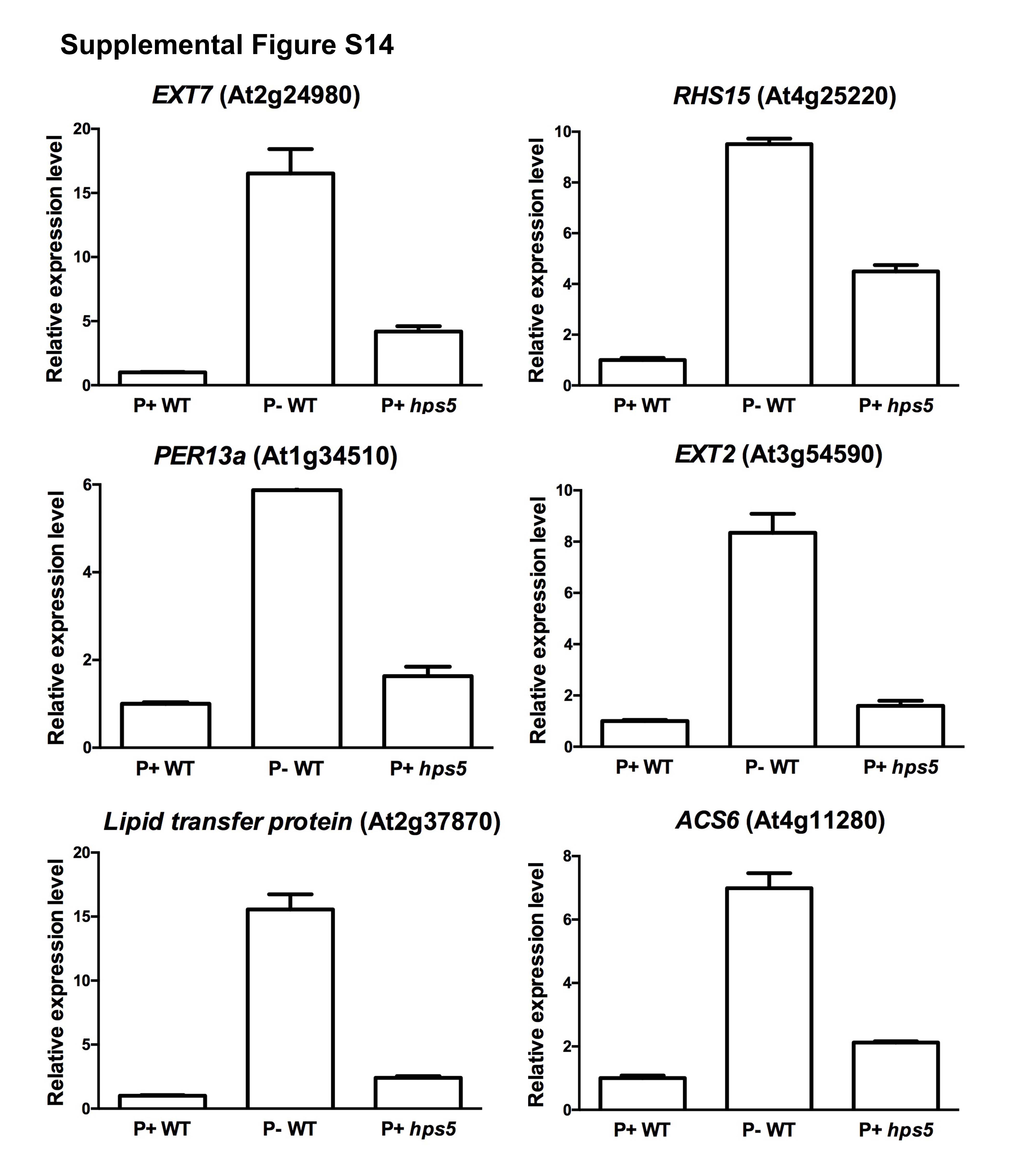

Supplement: S14 Fig — (JPG) [file pgen.1006194.s014.jpg]

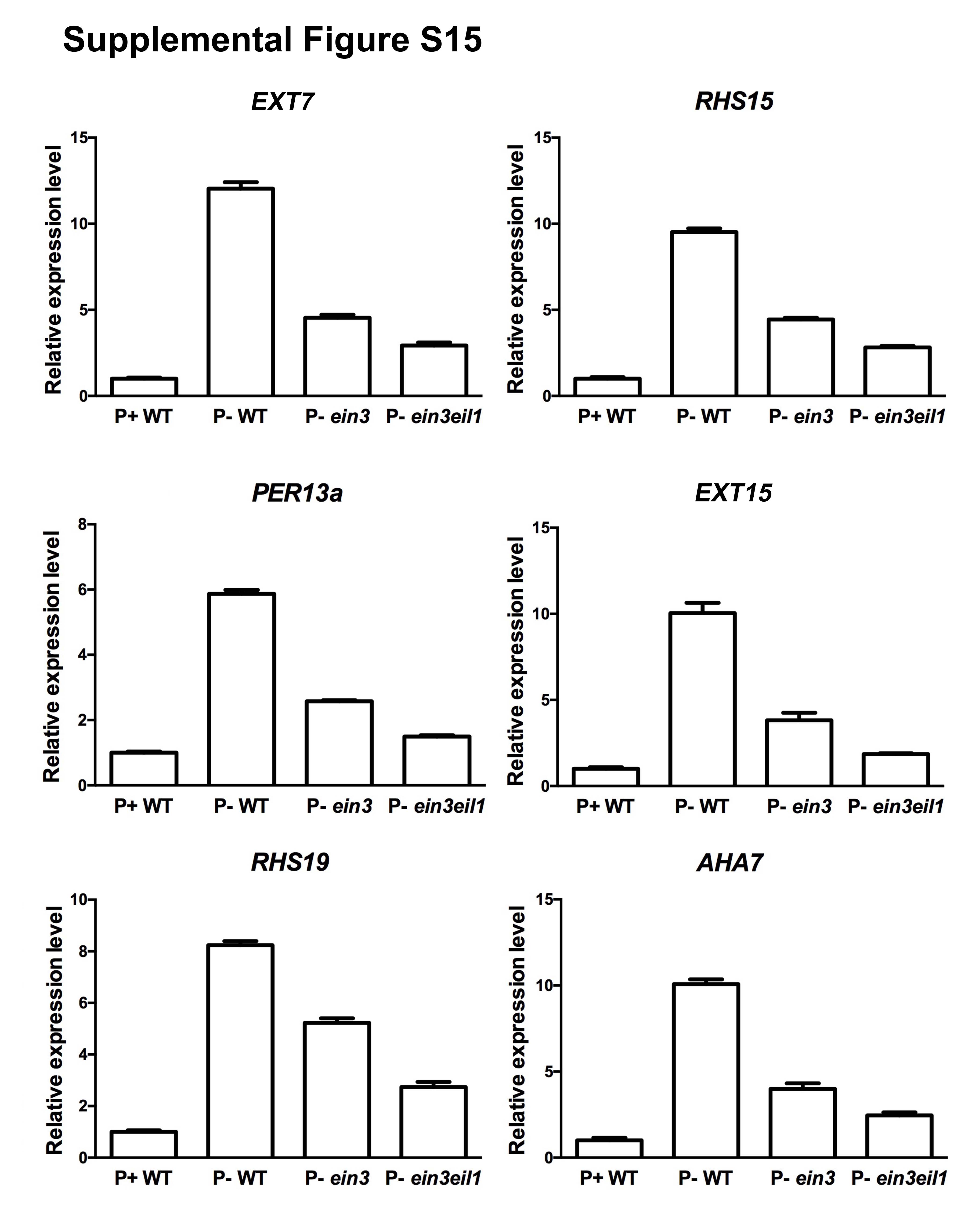

Supplement: S15 Fig — (JPG) [file pgen.1006194.s015.jpg]

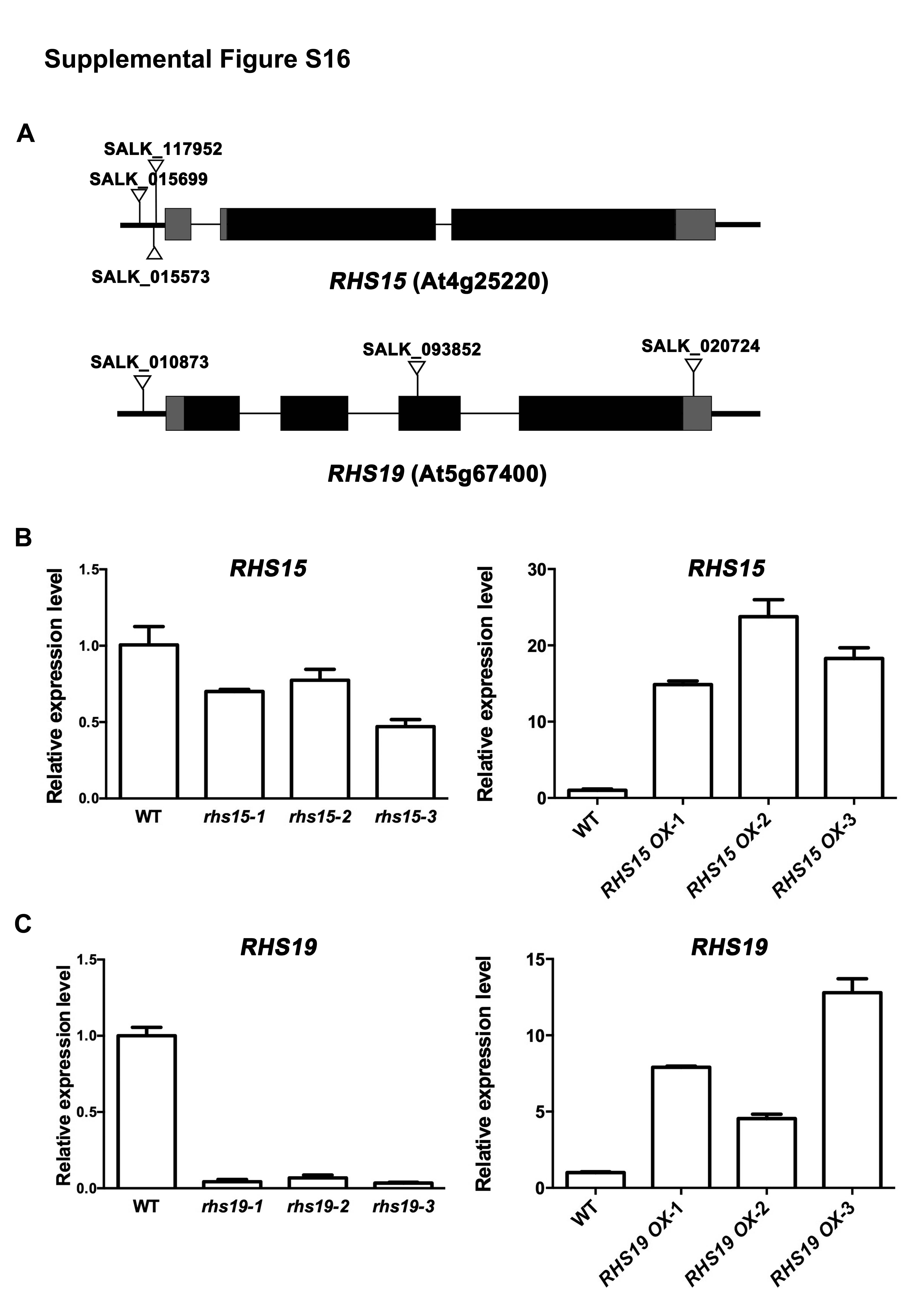

Supplement: S16 Fig — (A) Diagrams showing the positions of the T-DNA in the RHS15 and RHS19 gene. Black box: exons; grey box: 5’ and 3’ UTRs; thin line: introns; thick line: flanking sequences surrounding the gene. Relative expression of RHS15 (B) and RHS19 (C) in the 7-day-old seedlings of their corresponding T-DNA insertion lines and overexpressing lines as determined by qPCR. The name of SALK lines for each allele of rhs15 and rhs19 are as follows. rhs15-1: SALK_015573; rhs15-2: SALK_015669; rhs15-3: SALK_117952. rhs19-1: SALK_093852; rhs19-2: SALK_010873; rhs19-3: SALK_020724. (JPG) [file pgen.1006194.s016.jpg]

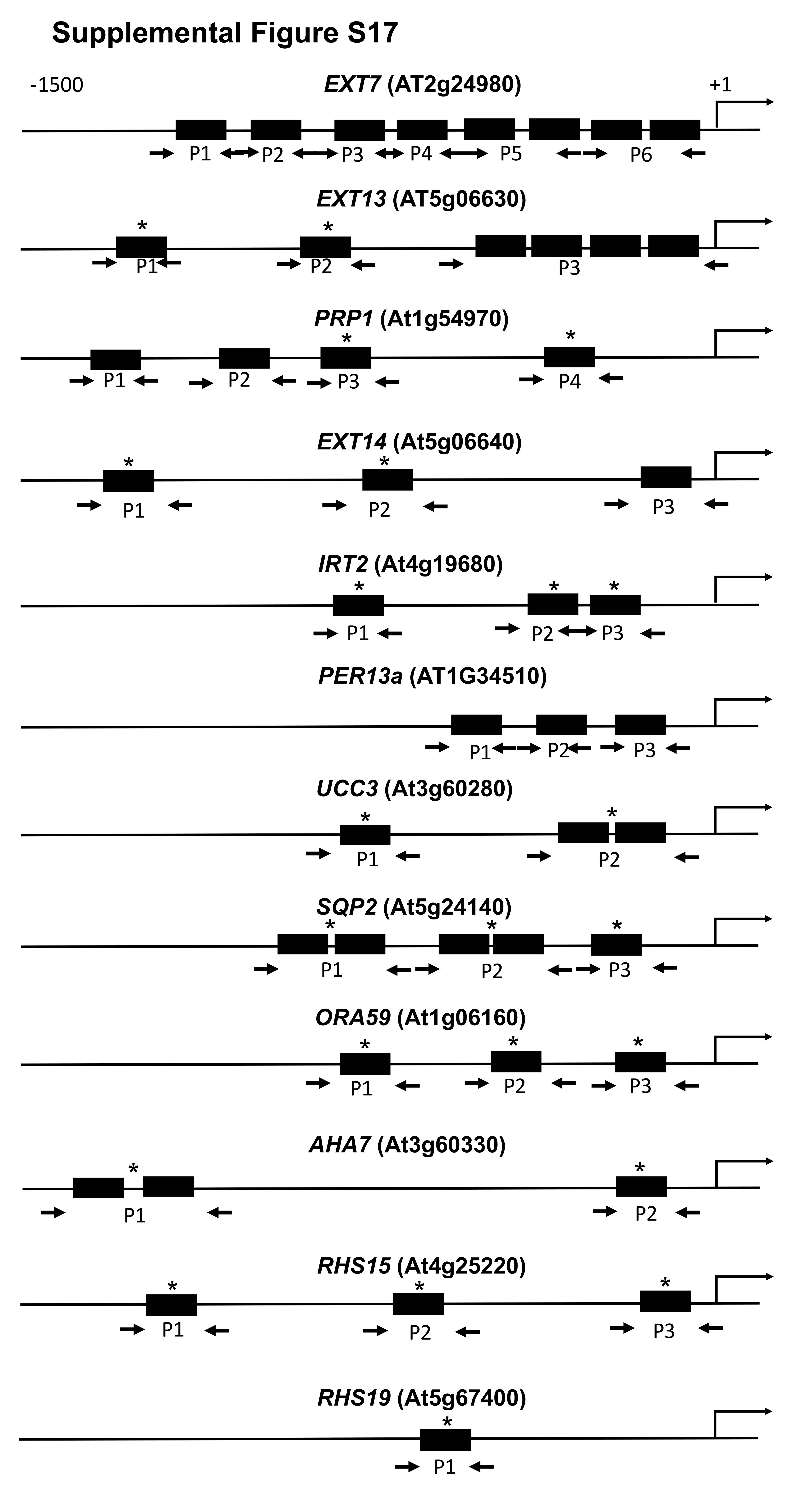

Supplement: S17 Fig — The 1.5 kb upstream sequences are shown, and the translational start sites (ATG) are shown at position +1. Arrows indicates the primers used for ChIP assays. The asterisks indicated the binding sites that is bound by EIN3 proteins. (JPG) [file pgen.1006194.s017.jpg]

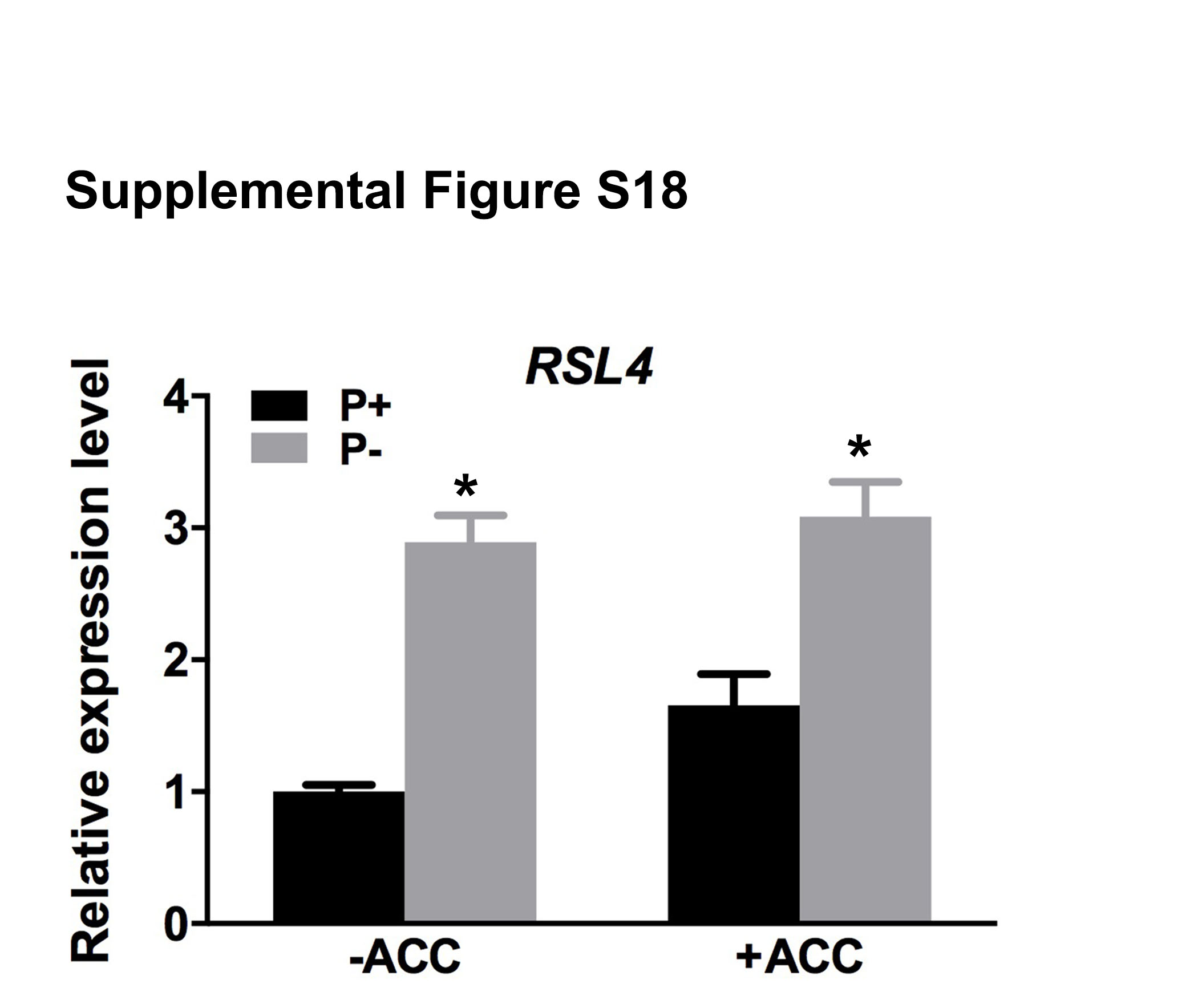

Supplement: S18 Fig — (JPG) [file pgen.1006194.s018.jpg]
